# On systematically building a controlled natural language for functional requirements

**DOI:** 10.1007/s10664-021-09956-6

**Published:** 2021-06-09

**Authors:** Alvaro Veizaga, Mauricio Alferez, Damiano Torre, Mehrdad Sabetzadeh, Lionel Briand

**Affiliations:** 1 SnT Centre for Security, Reliability and Trust, University of Luxembourg, Luxembourg City, Luxembourg; 2 School of Electrical Engineering and Computer Science, University of Ottawa, Ottawa, Canada

**Keywords:** Natural language requirements, Functional requirements, Controlled natural language, Qualitative study, Case study research

## Abstract

Natural language (NL) is pervasive in software requirements specifications (SRSs). However, despite its popularity and widespread use, NL is highly prone to quality issues such as vagueness, ambiguity, and incompleteness. Controlled natural languages (CNLs) have been proposed as a way to prevent quality problems in requirements documents, while maintaining the flexibility to write and communicate requirements in an intuitive and universally understood manner. In collaboration with an industrial partner from the financial domain, we systematically develop and evaluate a CNL, named Rimay, intended at helping analysts write functional requirements. We rely on Grounded Theory for building Rimay and follow well-known guidelines for conducting and reporting industrial case study research. Our main contributions are: (1) a qualitative methodology to systematically define a CNL for functional requirements; this methodology is intended to be general for use across information-system domains, (2) a CNL grammar to represent functional requirements; this grammar is derived from our experience in the financial domain, but should be applicable, possibly with adaptations, to other information-system domains, and (3) an empirical evaluation of our CNL (Rimay) through an industrial case study. Our contributions draw on 15 representative SRSs, collectively containing 3215 NL requirements statements from the financial domain. Our evaluation shows that Rimay is expressive enough to capture, on average, 88% (405 out of 460) of the NL requirements statements in four previously unseen SRSs from the financial domain.

## Introduction

Requirements are considered as one of the fundamental pillars of software development. For many systems in industry, requirements are predominantly expressed in natural language (NL). Natural language is advantageous in that it can be used in all application domains and understood virtually by all project stakeholders (Pohl 2010). Supporting this statement, studies reported that 71.8% of software requirements specifications (SRSs) are written in NL (Mich et al. 2004) and that the majority of users (61%) prefer to express requirements using NL (Kassab et al. 2014). Furthermore, Zhao et al. (2020) posit that NL will continue to serve as the *lingua franca* for requirements in the future. Despite its pervasive use, undisciplined use of NL can bring about a variety of quality issues. Common problems with NL requirements include: poor testability, inappropriate implementation, wordiness, under-specification, incompleteness, duplication, omission, complexity, vagueness, and ambiguity (Mavin and Wilkinson 2010; Fernȧndez et al. 2017).

Further, requirements often change throughout a project’s lifespan until a consensus is reached among stakeholders. Requirements changes lead to significant additional costs that vary according to the project phase (Hull et al. 2011); it has long been known that the cost of fixing problems related to requirements increases rapidly when progressing through the software development phases (Boehm and Basili 2001).

The ultimate quality of a software system greatly depends on the quality of its requirements. Empirical evidence shows that the state of practice for acquiring and documenting requirements is still far from satisfactory (Sadraei et al. 2007; Solemon et al. 2009; Young 2015). Different studies have reported that one of the main causes of software project failures in industry is related to poorly written requirements, i.e., requirements that are unclear, ambiguous, or incomplete (Ahonen and Savolainen 2010; Hull et al. 2011; The Standish Group 1995). Poorly written requirements are difficult to communicate and reduce the opportunity to process requirements automatically, for example, to extract models (Arora et al. 2015) or derive test specifications (Alférez et al. 2019).

The problem we address in this article was borne out of a practical need observed across many industrial domains. For example, in the financial domain, the current practice is to write system requirements using a general-purpose text editor without enforcing any requirement structure. This is the case for our industrial partner, Clearstream Services SA Luxembourg – a post-trade services provider owned by Deutsche Borse AG. Clearstream reported that several communication problems and delays arise from requirements that are not stated precisely enough, particularly in situations where the project development tasks are divided across several teams in different countries. This problem is compounded by the fact that Clearstream typically has to deal with SRSs written in NL that are created by domain experts (from now on, we refer to them as “financial analysts”), who do not necessarily possess sufficient expertise in requirements elicitation and definition.

Furthermore, other stakeholders at different levels of the organization, e.g., customer service, also need to be able to process the requirements and validate them according to their specific needs (Dick et al. 2017). As a result, there is a tension between the pressure to use NL in practice and the need to be more precise and resorting to formal languages (Yue et al. 2011). Controlled natural languages (CNLs) strike a balance between the usability of NL on the one hand and the rigour of formal methods on the other. A CNL is a set of predefined sentence structures that restrict the syntax of NL and precisely define the semantics of the statements written using these predefined structures (Pohl 2010).

In this article, we concern ourselves with developing a CNL for writing requirements for financial applications. We have named our CNL Rimay, which means “language” in Quechua. We focus on *functional requirements*, noting that the vast majority of the requirements written by our industrial partner are functional, and that financial analysts find most of the ambiguity and imprecision issues in functional requirements. The functional requirements produced by Rimay are intended to replace unrestricted requirements and, as a result, enable the automation of certain tasks, such as the generation of acceptance-test criteria (Veizaga et al. 2020). In our context, a functional requirement specifies what system response an actor is expected to receive when providing certain inputs, if certain conditions are met. We consider every other type of requirement to be non-functional.

While Rimay is grounded in requirements for financial applications, it also overlaps with other Requirements Engineering ontologies such as the Core Ontology for REquirements (CORE) (Jureta et al. 2009), whose development was inspired by the work of Zave and Jackson (1997). In short, CORE aims to cover all the basic concerns that stakeholders communicate during the requirements elicitation process (beliefs, desires, intentions, and evaluations) by introducing a set of concepts (Goal, Plan, Domain assumption, and Evaluation). Each concept, except Plan, has subcategories. For instance, the goal concept has three subcategories in CORE: Functional goal, Quality constraint, and Softgoal. The condition structure and system response of Rimay correspond to the Functional goal concept of CORE.

Finally, although our work draws on the requirements of financial applications, this domain shares several common characteristics with other domains where (data-centric) information systems are developed. We therefore anticipate that our results, including our methodology, lessons learned, and Rimay itself, can be a useful stepping stone for building CNLs in other related domains. This said, we acknowledge that additional empirical work remains necessary to substantiate claims about usefulness beyond our current domain of investigation, i.e., finance.

Our investigation is guided by the following research questions (RQs):

- **RQ1: What information content should one account for in the requirements for financial applications?** In this RQ, we want to identify, in the requirements provided by our industrial partner, the information content used by financial analysts. This information is a prerequisite for the design of the Rimay grammar.
- **RQ2: Given the stakeholders, how can we represent the information content of requirements for financial applications?** After we identify the information content used by our industrial partner to represent requirements, we want to find out the structures of the requirements that our CNL should support. These structures follow recommended syntactic structures and define mandatory and optional information.
- **RQ3: How well can Rimay express the requirements of previously unseen documents?** After building our CNL grammar, we need to determine how well it can capture requirements in unseen SRSs.
- **RQ4: How quickly does Rimay converge towards a stable state?** Rimay reaches a stable state when it does not need to continuously evolve (i.e., no addition of new rules and no updates to the existing rules) in response to the analysis of new (unseen) SRSs. To assess stability, we use the notion of saturation. Saturation occurs, in a qualitative study, when no new information seems to emerge during coding.

In this article, we use a total of 15 SRSs written by financial analysts at Clearstream. These SRSs describe different projects that cover a range of activities: nine discuss the updating of existing applications, two concern the compliance of the applications with new regulations, two describe the creation of new applications, and the last two describe the migration of existing applications to more sophisticated technologies. Of the 15 SRSs, 11 are used in our qualitative study to answer RQ1 and RQ2, and the other four in our empirical evaluation to answer RQ3 and RQ4.

We use a combination of Grounded Theory and Case Study Research to address the four research questions posed above. The main contributions of this work can be summarized as follows:

1. A qualitative methodology aimed at defining a CNL for functional requirements (RQ1). We rely on Grounded Theory for developing Rimay. Grounded Theory is a systematic methodology for building a theory from data. The goal of Grounded Theory is to generate theory rather than test or validate an existing theory (Stol et al. 2016). Our methodology is general and can serve as a good guiding framework for building CNLs systematically. We rely on an analysis procedure named *protocol coding* (Saldaña 2015), which aims at collecting qualitative data according to a pre-established theory, i.e., set of codes. Protocol coding allows additional codes to be defined when the set of pre-established codes is not sufficient. A code in qualitative data analysis is most often a word or short phrase that symbolically assigns a summative, salient, essence-capturing, and/or evocative attribute for a portion of language-based or visual data (Saldaña 2015). In the context of our article, a code identifies a group of verbs that share the same information content in an NL requirement. As explained in Section 3.3.2, most of the codes are pre-existing verb-class identifiers available in a well-known lexicon named VerbNet^1^. In addition, we use WordNet^2^ to verify the verb senses of the requirements. The fact that we use domain-independent lexical resources and include no keywords specific to the financial domain in Rimay, makes our approach more likely to have wider applicability to information systems in general. We conduct our qualitative study on 11 SRSs that contain 2755 requirements in total.
2. A CNL grammar (RQ2) targeting financial applications in particular and information systems in general. We apply restrictions on vocabulary, grammar, and semantics. The Rimay grammar accounts for a large variety of system responses and conditions, while following recommended syntactic structures for requirements (e.g., the use of active voice). Also, the Rimay grammar defines mandatory information content to enforce the completeness of functional requirements. In addition to the grammar, we generate a user-friendly and full-featured editor using the language engineering framework Xtext.^3^
3. An empirical evaluation of Rimay (RQ3 and RQ4). We report on a case study conducted within the financial domain. We evaluate Rimay on four SRSs containing 460 requirements to demonstrate the feasibility and benefits of applying Rimay in a realistic context. We use *saturation* to find the point in our evaluation where enough SRS content has been analyzed to ensure that Rimay is stable for specifying requirements for the financial domain. Furthermore, we use a *z-test for differences in proportions* to confirm that additional enhancements to Rimay are unlikely to bring significant benefits.
  The article is structured as follows: Section 2 introduces the background and related work. Section 3 presents a qualitative study aimed at analyzing the information content in the requirements provided by Clearstream (our industrial partner). In Section 4, we describe the details of Rimay. Section 5 describes a case study that evaluates Rimay. Threats to the validity of our results are discussed in Section 6. Section 7 discusses practical considerations and, finally, our conclusions and an outline of future work are provided in Section 8.

## Background and related work

This section reviews the lexical resources we rely on in this work and further discusses related work.

### Lexical resources

In the next subsections, we discuss WordNet and VerbNet. We use the WordNet dictionary for verb lookup operations and the VerbNet lexicon to cluster verbs with similar semantics into verb classes.

#### WordNet

WordNet (Miller 1995) is a domain-independent linguistic resource which provides, among several other things, more than 117000 *synsets*. Synsets are synonyms –words that denote the same concept and are interchangeable in many contexts– grouped into sets. Each synset contains (a) a brief definition (“gloss”), (b) the synset members, and, in most cases, (c) one or more short sentences illustrating the use of the synset members. Each synset member is a synonym sharing the same sense of the other members of the synset. Synset members use the format *word#sense* *number*. For example, in WordNet the verb *create* has six synsets. One of those synsets contains the following information: (a) gloss, “create or manufacture a man-made product”, (b) two synset members, *produce#2* and *make#6*, and (c) an example of how to use the synset members *produce#2* and *make#6*, “We produce more cars than we can sell”.

In order to develop Rimay, in Section 3.3.2, we use WordNet to retrieve the different synonyms and senses of the verbs identified in the NL requirements.

#### VerbNet

VerbNet (Kipper et al. 2000) is a domain-independent, hierarchical verb lexicon of approximately 5800 English verbs. It clusters verbs into over 270 verb classes, based on their shared syntactic behaviors. Each verb in VerbNet is mapped to its corresponding synsets in WordNet, if the mapping exists. In VerbNet, a verb is always a member of a verb class and each verb class is identified by a unique code composed of a name and a suffix. The suffix reveals the hierarchical level of a verb class, e.g., two of the sub-classes of the root class *multiply-108* are *multiply-108-1* and *multiply-108-2*. In VerbNet, the sub-classes inherit features from the root class and specify further syntactic and semantic commonalities among their verb members. For example, each of the sub-classes of *multiply-108* uses the same syntactic structure which is defined as a noun phrase followed by a verb, a noun phrase, and a prepositional phrase. However, each sub-class uses different prepositions in the prepositional phrase. In particular, the subclass *multiply-108-1* has the verb members *divide* and *multiply* and uses the preposition *by* as in the phrase “I multiplied x by y”. The subclass *multiply-108-2* has verb members such as *deduct*, *factor*, and *subtract* and uses the preposition *from* as in the phrase “I subtracted x from y”.

In Section 3.3.2, we describe how we used VerbNet to identify the verb classes of the verbs that we found in our NL requirements.

### Related work

Numerous studies have been conducted with a focus on NL requirements quality improvement. Pohl (2010) presents three common techniques for improving the quality of NL requirements by reducing vagueness, incompleteness and ambiguity:

- **Glossaries**. Requirements glossaries make explicit and provide definitions for the salient terms in a SRS. Requirements glossaries may further provide information about the synonyms, related terms, and example usages of the salient terms (Arora et al. 2017).
- **Patterns**. They are pre-defined sentence structures that contain optional and mandatory components. Patterns restrict the syntax of the text and are meant to help stakeholders in writing more standardized NL requirements and thus circumventing frequent mistakes.
- **Controlled natural languages**. They are considered an extension of the pattern category which, in addition to restricting the syntax (the grammatical structures), also provide language constructs with which it is possible to precisely define the semantics of NL requirements.

In this article, we build a CNL to represent functional requirements in the financial domain. However, given that Rimay does not rely on any domain-specific constructs (Sections 2.1 and 3), it could also be applied to other (data-centric) information systems in different domains.

Given our objective, we focus here on approaches and studies related to CNLs and patterns for expressing NL requirements. We searched relevant approaches and studies in four well-known digital libraries: ACM, IEEE, Springer, and ScienceDirect. In addition, we considered relevant surveys that discuss CNLs and patterns for expressing NL requirements. We selected 11 studies, directly relevant to our work, that focus on improving NL requirements through the use of patterns or CNLs. Table 1 outlines the main characteristics of these studies. The first column of the table provides a reference to each study. The second column indicates the type of the approach, i.e., Pattern or CNL. In order to obtain a more thorough picture of the literature, although our work is focused on functional requirements, our analysis of the related work does not exclude references that exclusively address non-functional requirements. The third column shows the type of the requirements that the approach supports: Functional Requirements (FR), Non-Functional Requirements (NFR), or both. Additionally, the third column includes the domain in which the patterns and CNLs were created. There are two strands of work: domain-independent and domain-specific (i.e., automotive, business, healthcare, performance, embedded systems, and data-flow reactive systems).

**Table 1:** Summary of related work

Study	Type of	Type of	Systematic	Evaluation	Tool
Reference	Approach	Requirements	Study		Support
Pohl and Rupp (2011)	Pattern	FR (Domain-Independent)	No	No	No
Mavin et al. (2009)	Pattern	FR (Domain-Independent)	No	Yes	Yes
Withall (2007)	Pattern	Both (Business)	No	No	No
Riaz et al. (2014)	Pattern	NFR (Healthcare)	No	No	Yes
Eckhardt et al. (2016)	Pattern	NFR (Performance)	Yes	Yes	No
Denger et al. (2003)	Pattern	FR (Embedded Systems)	No	Yes	No
Konrad and Cheng (2005b)	CNL	NFR (Automotive)	No	Yes	Yes
Fuchs et al. (2008)	CNL	Both (Several)	No	No	Yes
Post et al. (2011)	CNL	FR (Automotive)	No	Yes	Yes
Crapo et al. (2017)	CNL	FR (Domain-Independent)	No	No	Yes
Carvalho et al. (2014)	CNL	Both (Data-Flow Reactive systems)	No	No	Yes

The fourth column indicates whether an empirical study was conducted and evaluated in a systematic manner. The fifth column shows whether the proposed CNL or pattern was somehow evaluated. Finally, the sixth column reports on whether tool support was provided.

We discuss the selected studies next.

#### Patterns

Pohl and Rupp (2011) discuss a single pattern to specify functional requirements. The authors claim that the requirements that comply to this pattern are explicit, complete and provide the necessary details to test such requirements.

Mavin et al. (2009) define the Easy Approach to Requirements Syntax (EARS), which is a set of five patterns enabling analysts to describe system functions. The authors demonstrate, through a case study in the aviation domain, that using EARS leads to requirements which are easier to understand and which exhibit fewer quality problems, particularly in relation to ambiguity. Tool support for the EARS patterns was presented in a follow-up paper (Lúcio et al. 2017).

Withall (2007) identifies 37 patterns to specify structured functional and non-functional requirements for the business domain. The study provides insights regarding the creation and extension of the patterns.

Riaz et al. (2014) define a set of 19 functional security patterns. They provide a tool that assists the user in selecting the appropriate pattern based on the security information identified in the requirements.

Eckhardt et al. (2016) propose patterns to specify performance requirements. The patterns were derived from a content model built from an existing performance classification. Eckhardt et al. (2016) define the content elements that a performance requirement must contain to be considered complete.

Denger et al. (2003) propose a set of patterns to describe requirements for embedded systems. The patterns were derived from a metamodel that captures several types of embedded-system requirements. The authors validate their patterns through a case study.

In contrast to the other four studies, Riaz et al. (2014) and Mavin et al. (2009) provide tool support to guide analysts in defining requirements. Eckhardt et al. (2016) follow a systematic process to develop a framework for the creation of performance requirements patterns, and presented a well-defined evaluation of their approach.

#### Controlled natural languages

Konrad and Cheng (2005b) provide a restricted natural language for the automotive and appliance domains, enabling analysts to express precise qualitative and real-time properties of systems. They evaluated their approach through a case study and introduced their tool in a follow-up paper (Konrad and Cheng 2005a).

The approach described by Fuchs et al. (2008) was identified from the survey and classification of CNLs conducted by Kuhn (2014).

Fuchs et al. (2008) propose the Attempto Controlled English, which is a CNL that defines a subset of the English language intended to be used in different domains, such as software specification and the Semantic Web. Attempto can be automatically translated into first-order logic.

Post et al. (2011) identify three new rules that extend the approach proposed by Konrad and Cheng (2005b) to express requirements in the automotive domain. They validated their rules through a case study, and described their tool in another paper (Post and Hoenicke 2012).

Crapo et al. (2017) propose the Semantic Application Design Requirements Language which is a controlled natural language in English for writing functional requirements. Their language supports the mapping to first-order logic. Carvalho et al. (2014) propose a CNL called SysReq-CNL that allows analysts to describe data-flow requirements. Their sentence rules are nonetheless not mapped onto any formal semantics. None of the above approaches have been empirically evaluated.

To summarize, no previous strand of work describes a systematic process to build CNL grammar rules. However, all the above approaches provide tool support to assist analysts with specifying requirements.

#### Differences between the related work and our approach

No other work, in our knowledge, follows a systematic process for creating and evaluating a CNL to specify functional requirements, either in the financial domain (the main focus of our investigation) or any other domain. More precisely, our work differs from the existing work in the following respects: (a) we derive Rimay from the analysis of a large and significant number of requirements from the financial domain; (b) we create Rimay by following a rigorous and systematic process; (c) we evaluate Rimay through a case study based on industrial data while following empirical guidelines for conducting Case Study Research (Runeson et al. 2012); and (d) we fully operationalize Rimay through a usable prototype tool.

## Qualitative study

In this section, we report on a qualitative study aimed at characterizing the information content found in the functional NL requirements provided by Clearstream. In the following, every time we speak of “requirements”, we mean functional NL requirements.

Other techniques, such as grammar induction (Stevenson and Cordy 2014), could have been used to learn the syntax of the functional requirements in an automated manner. However, we believe that the limited number of available requirements would not have resulted in a reliable learning model. Therefore, we opted to conduct a qualitative study to build a semi-automated strategy enabling the creation of the grammar rules in a precise manner.

First, we describe the context of the qualitative study along with the criteria used to select SRSs. Then, we present the analysis procedure of our qualitative study where we show the codes that identify different groups of requirements. Each group of requirements is characterized by different information content. In this work, information content refers to the meaning assigned to the text of the requirements.

The result of the analysis procedure is a grammar that defines the syntax of a CNL that is able to specify all the information content found in the analyzed requirements. A grammar is a set of controlled and structured syntax rules (also known as grammar rules) describing the form of the elements that are valid according to the language syntax (Bettini 2013). In our context, our grammar controls the structure of functional requirements by applying syntax rules. Section 3.3.5 (Step 5.2) describes how we produce the Rimay grammar rules, and Section 4 describes all the grammar rules of Rimay.

### Research question

The goal of this qualitative study is to answer the following research question: RQ1: What information content should one account for in the requirements for financial applications? RQ1 aims to identify the mandatory and optional information content used by Clearstream to describe requirements. This is essential in order to design a CNL that will help financial analysts write requirements that are as complete and as unambiguous as possible.

### Study context and data selection

We conducted this study in collaboration with Clearstream Services SA Luxembourg, which is a securities services company with 2500 customers in 110 countries. More concretely, we worked with the Investment Fund Services (IFS) division. An Investment Fund is a capital that belongs to a number of investors and is used to collectively invest in stocks and bonds. Among other tasks, the IFS division takes care of (a) the development of new applications, (b) upgrading existing ones, and (c) the migration of applications to more sophisticated technologies to provide their clients with state-of-the-art solutions that comply with the regulations in force. The Clearstream units involved with IFS are project management, IFS and market operations, design, functional and business analysis, development, and testing.

Clearstream performs the aforementioned tasks following a methodology grounded in best practices and years of experience. For instance, financial analysts specify requirements using a combination of UML models and natural language requirements following the Rupp template (Pohl and Rupp 2011). Clearstream follows a carefully planned software development process (Sommerville 2011) based on the V-Model, that is suitable for a heavily regulated industry, such as finance.

Clearstream is continuously delivering new software projects in the financial domain and employs English as the primary language for specifying requirements. Two members of our research team were embedded in the Clearstream - IFS to get familiar with the company’s development process and its organizational culture for over a month before starting the project described in this article. Our members participated in training sessions and numerous meetings organized by Clearstream. Additionally, all the research team members have been interacting, both electronically and through face-to-face meetings, with the members of the IFS team for two years.

We validated our results and conclusions with a team of experts. The team was composed of eight financial analysts: (a) two were *senior* financial analysts with more than 20 years of experience in specifying requirements in the financial domain. Their areas of expertise are business analysis, functional design, functional architecture, requirements engineering, and project management; (b) Four of them were *mid-career* financial analysts with more than 10 (but less than 20) years of experience in business and functional analysis in the financial domain. One of the *mid-career* analyst had software programming and testing skills; and (c) two were *junior* financial analysts with two to five years of experience in business analysis. This validation activity was performed over a year in an iterative and incremental manner with face-to-face, bi-weekly sessions with the team of experts, with each of these sessions lasting between two to three hours. This activity was concluded when the experts did not have any additional suggestions for improving the clarity, completeness, or correctness of the requirements.

Among all those available in Clearstream, we selected SRSs which: (a) belong to recently concluded projects, (b) contain at least 15 requirements, (c) contain requirements written in English, and (d) are written by different financial analysts. The senior financial analysts from Clearstream selected 11 representative SRSs according to the four criteria defined above. Each one of the SRSs contained the following types of information: business context, goals and objectives, project scope, current and future overview, general information (e.g., glossary, related documentation, acronyms and abbreviations), and Unified Modeling Language (UML) diagrams for the high-level functional decomposition of the systems and requirements. In total, the 11 SRSs contained 2755 requirements.

### Analysis procedure

**Fig. 1:**
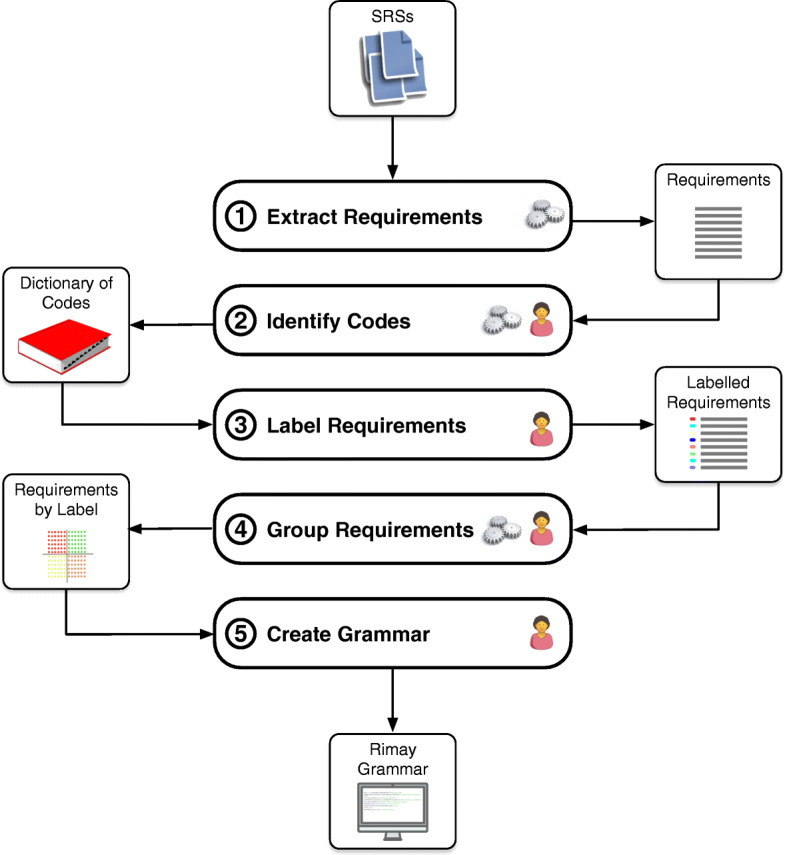
Overview of our analysis procedure

Figure 1 shows an overview of our semi-automated analysis procedure. In Step 1, we first extracted 2755 requirements from 11 SRSs. In Step 2, we identified a dictionary of 41 codes from the extracted requirements. For example, the code *send_11.1* identifies five verbs used in the extracted requirements: “return”, “send”, “forward”, “pass”, “export” and “import”(Tables 4 and 5 shows the 41 codes and verbs identified in our qualitative study and the evaluation). Our analysis procedure for identifying the codes followed *protocol coding* (Saldaña 2015), which is a method for collecting qualitative data according to a pre-established theory, i.e., a set of codes. As explained later in this section, our pre-established set of codes was identified from VerbNet. Using a coding system based on a predefined set of codes helps us to save analysis time and mitigate coding bias. In Step 3, two annotators (first and second authors of this article) labeled the extracted requirements with one or more of the codes discovered in the previous step. In Step 4, we grouped the extracted requirements by their labels. The purpose of grouping requirements is to ease the identification of common information content to create grammar rules. For example, all the requirements that use the verbs members of the code *send_11.1* share the semantic roles INITIAL LOCATION (a place where an event begins or a state becomes true) and DESTINATION (a place that is the end point of an action and exists independently of the event). In Step 5, we iteratively created and integrated the grammar rules into Rimay. Each of the five steps in Fig. 1 shows one or two icons denoting whether a given step was carried out (1) automatically (i.e., the three gears icon), (2) manually (i.e., the human icon), or (3) semi-automatically (i.e., both icons).

The next subsections describe in details Steps 1 to 5.

#### Extract requirements (Step 1)

We read the 11 SRSs and extracted 2755 requirements. In our case, all the requirements were written in tables in which all the requirements were clearly identified and distinguished from other information. The structure of the SRSs clearly separates functional from non-functional requirements. Furthermore, we checked that no functional requirement was mistakenly placed in the non-functional requirements section. We verified that the content of the requirements presenting lists and tables was correctly captured by our automatic extraction algorithm. If there was any error, we manually corrected it. This step was automated using the Apache POI API,^4^ which is a well-known Java library for reading and writing files in Microsoft Office formats.

Table 2 shows three requirements extracted from a SRS. The column “Id” identifies the requirements, the column “Description” contains the original text of the requirements, and the column “Rationale” presents the reasoning behind the creation of a given requirement.

**Table 2:** Three requirements extracted from a SRS during Step 1 of Fig. 1

Id	Description	Rationale
TNG.INPUT.010	If the message contains “FISN”, then the System must ignore the message.	FISN is an official ISO Standard created to enhance the quality of financial messaging.
TRAN.0030	The System must regenerate the outbound XML according to the new XML specification “SR2017”.	The previously created orders, which their status are activated, must be changed to comply with the new XML specification.
Data.SAA.060	The data of the System older than 13 months must be archived for at least 10 years.	This requirement complies to a legal rule.

#### Identify codes (Step 2)

The coding approach is intended to (1) obtain a number of codes that allow the language to be expressive enough for the financial domain, (2) be systematic to allow others to replicate the procedure, and (3) ensure that Rimay remains as broadly applicable as possible by minimizing reliance on domain-specific terms. The requirements specify the expected system behavior using verb phrases, e.g., “*send* a message” and “*create* an instruction”. We used the verb lexicon named VerbNet (Section 2.1.2) to identify the codes from our SRSs. Section 3.3.5 will explain in details how, by using verb classes, we obtain the grammar rules of Rimay.

We followed a semi-automated process to identify codes and their corresponding verbs. We automated some of the sub-steps of Step 2 by using the NLTK^5^ library for Python. In the remainder of this section, we describe in detail which sub-steps of Step 2 were automated. From the 41 codes that we proposed in this qualitative study, 32 codes (78%) correspond to verb class ids from VerbNet (referred to thereafter as *VerbNet codes*), and nine (22%) are codes that we proposed because they were missing from VerbNet but were needed to analyze the requirements. We use below the following terms to describe this process:

- REQS: Set of requirements to analyze.
- LEMMAS: List of lemmas found in the action phrases of REQS.
- CODES: Dictionary of codes and their corresponding verb members found during our analysis procedure. There are two types of codes: VerbNet codes and codes proposed by us.
- AUX: Auxiliary list of the lemmas that are not members of any code in CODES.
- SYNS: Dictionary of lemmas and their corresponding applicable synonyms.
- VN: Read-only dictionary of all the publicly available VerbNet codes and their corresponding verb members.

**Fig. 2:**
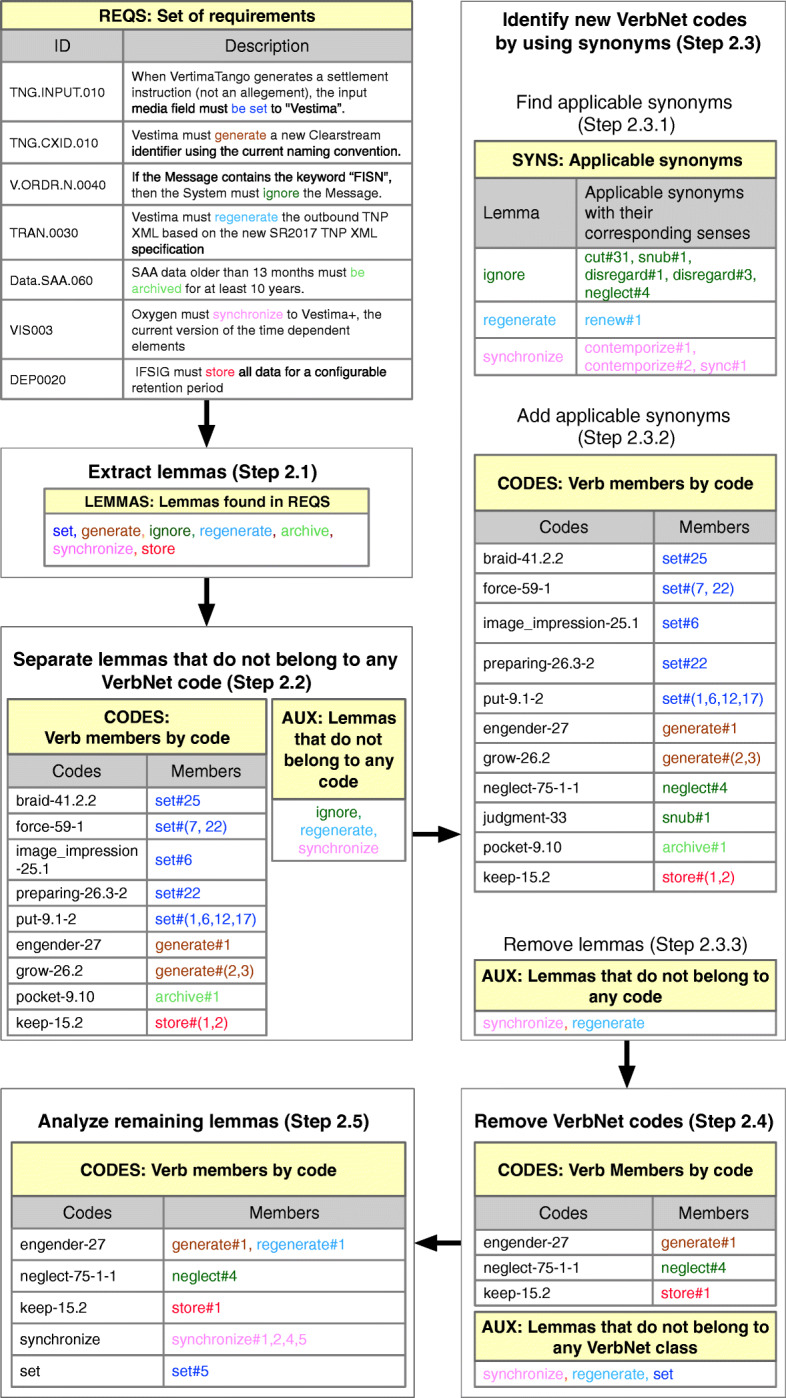
Identify codes (Step 2)

In Fig. 2, we show a running example of our process to identify the codes. The process steps are as follows:

##### Extract lemmas (Step 2.1)

We extracted the verbs of each requirement in REQS (upper-left corner of Fig. 2) to obtain lemmas. A lemma is the base form of the verb. For example, from “archived”, the lemma is “archive”. We stored the resulting lemmas in LEMMAS.

##### Separate lemmas that do not belong to any VerbNet code (Step 2.2)

We retrieved for every lemma in LEMMAS its corresponding VerbNet codes from VN. We stored these VerbNet codes and their corresponding lemmas (including their sense number, depicted as a number after the symbol *#*) in CODES. For example, the key-value pair *{engender-27, generate#1}* in CODES of Fig. 2 (Step 2.2) means that the lemma *generate* (Step 2.1 of Fig. 2) with the sense number one (i.e., “bring into existence”) is a member of the VerbNet code *engender-27*.

If a lemma in LEMMAS was not a member of any VerbNet code in VN, we added it to an auxiliary list of lemmas named AUX. For example, in Fig. 2 (Step 2.2) we added to AUX the lemmas *ignore*, *regenerate* and *synchronize* that were not identified in VN, but were found in the analyzed requirements.

##### Identify new VerbNet codes by using synonyms (Step 2.3)

We analyzed the synonyms and senses of the lemmas in AUX to discover new VerbNet codes that can be added to CODES. We describe this process in more details as follows:

##### Find applicable synonyms (Step 2.3.1)

We used WordNet to retrieve all the synonyms of each auxiliary lemma in AUX. We stored in SYNS only the synonyms whose senses match the sense of an auxiliary lemma as used in REQS.

As an example, Table 3 shows the list of synonyms of the lemma *regenerate*, which is one of the lemmas in AUX shown in Fig. 2 (Step 2.2). The synonyms in Table 3 are grouped according to the sense numbers of the lemma *regenerate*, namely 1, 3, 4 and 9 (according to WordNet, the verb *regenerate* has nine senses, but Table 3 only shows the senses that have at least one synonym). From the four senses in Table 3, we chose the ones that match the sense of the verb *regenerate* used in REQS. In this case, we chose sense number 1 since it was the only sense that was applicable to the requirements. Finally, we store in SYNS the synonyms and their chosen sense numbers. In the case of the lemma *regenerate*, we only added *renew#1* to SYNS.

**Table 3:** Senses and synonyms of the verb *regenerate* retrieved from WordNet

Sense	Sense Definition	Synonyms and	Chosen
Number		Their Sense Number	Sense?
1	Reestablish on a new, usually improved, basis or make new or like new	renew#1	Yes
3	Bring, lead, or force to abandon a wrong or evil course of life, conduct, and adopt a right one	reform#2, reclaim#3, rectify#3	No
4	Return to life, get or give new life or energy	restore#2, rejuvenate#4	No
9	Restore strength	revitalize#1	No

##### Add applicable synonyms (Step 2.3.2)

We retrieved, for every synonym in SYNS, its corresponding VerbNet codes from VN. Then, we stored the retrieved VerbNet codes and the corresponding synonym (including the sense number) in CODES. For example, given that the synonym *neglect* (Step 2.3.1 of Fig. 2) with sense number four (i.e., *neglect#4*) is a member of the VerbNet code *neglect-75-1-1*, we created the key-value pair *{neglect-75-1-1, neglect#4}* in CODES (Step 2.3.2 of Fig. 2). If none of the synonyms of a lemma is a member of any code in VN, then we move the lemma from SYNS to AUX. For example, if the synonym is *renew#1* and it is not a member of any VerbNet code in VN, if it is a synonym of *regenerate* we then move *regenerate* from SYNS to AUX.

##### Remove VerbNet codes (Step 2.4)

In this step, our goal is to remove the VerbNet codes (from CODES) that are either not relevant to the SRSs in the financial domain or redundant. We performed this step during several offline validation sessions. Each session was attended by three to four financial analysts with the presence of at least one senior and one mid-career financial analyst.

At the end of Step 2.4 (Fig. 2), we went from 11 to three VerbNet codes (i.e., a reduction of 72,7%). Considering all the VerbNet codes used during this qualitative study, not only the 11 VerbNet codes shown in Step 2.4 in Fig. 2, we decreased the number of VerbNet codes from 158 to 32 (i.e., a reduction of 79,7%). The two strategies that we employed to reduce VerbNet codes are as follows:

- Strategy 1. Discard redundant verbs. For example, between the verbs *archive* and *store*, we discard the verb *archive* because the verb *store* is more frequent and both verbs are semantically similar.
- Strategy 2. Discard verbs that do not have applicable senses. For example, the VerbNet code *image_impression-25.1* (Step 2.3.2 of Fig. 2) involves only the member *set#6* whose sense is defined by WordNet as: “a relatively permanent inclination to react in a particular way”. Since this latter sense is not used in REQS, we finally discarded *image_impression-25.1* from CODES. After applying this strategy, if a verb was discarded from CODES, we added only its lemma to AUX for further manual analysis as we explain next in Step 2.5. For example, given that the verb *set* was discarded from CODES, we added its lemma (e.g., only the word *set* without sense*#*) to AUX.

##### Analyze remaining lemmas (Step 2.5)

In this step, we manually checked in WordNet if the senses of the remaining lemmas in AUX could be included in CODES. This step was carried out with the help of two senior and two mid-career financial analysts from Clearstream. We updated CODES when we identified an appropriate sense in WordNet that referred to one of the remaining lemmas. For example, in Fig. 2, we created the code *set* with a member *set#5* whose sense is used in REQS, and updated the VerbNet code *engender-27* with the member *regenerate#1*.

##### Coding results

Tables 4 and 5 present the resulting codes identified during our qualitative study described in Section 3.3.2 (“Identify Codes” (Step 2)). We finally obtained 41 codes, where 32 were obtained from VerbNet and nine were proposed by us.

**Table 4:** VerbNet codes identified during our qualitative study

Codes		Members
Class name	Hierarchy	
	Level	
admit	65	exclude
advise	37.9-1	instruct
allow	64.1	allow, authorize
beg	58.2	request
begin	55.1-1	begin
concealment	16-1	hide
contribute	13.2	restore
create	26.4	compute, publish
enforce	63	enforce
engender	27	create, generate
exchange	13.6	replace
forbid	67	prevent
herd	47.5.2	aggregate
involve	107	include
keep	15.2	store
limit	76	limit, restrict, reduce
mix	22.1-2	add
mix	22.1-2-1	link
neglect	75-1-1	neglect, ignore
obtain	13.5.2	accept, receive, retrieve
other_cos	45.4	close
put	9.1	insert
reflexive appearance	48.1.2	display, show
remove	10.1	extract, remove, delete
say	37.7-1	report, propose
see	30.1-1	detect
send	11.1	return, send, forward, pass
shake	22.3-2-1	concatenate
throw	17.1	discard
transcribe	25.4	copy
turn	26.6.1	convert, change, transform
use	105	apply
Total: 32

**Table 5:** Codes proposed during the qualitative study

Codes	Members
cancel	cancel
enable disable	enable, disable
get from	download
interrupt	interrupt
migrate	migrate
select unselect	select, unselect
synchronize	synchronize
update	update
validate	validate, check
Total: 9	

Table 4 provides the 32 VerbNet codes and their members. The first column of the table lists the codes, where each code is composed of a class name and a hierarchy level (Section 2.1.2). The second column shows the verb members related to the code. Table 5 shows the nine codes that we proposed. The first column of the table lists the codes and the second column provides the verb members associated to the code.

#### Label requirements (Step 3)

In Step 3 (Fig. 1), two annotators (the two first authors of this article) manually labeled the requirements extracted in Step 1 with one or more of the codes identified in Step 2. The labeling process required to (a) read the requirements and identify the verbs used in the system response of the requirements, (b) attempt to match the identified verbs with members of the codes found in Step 2, and (c) when there is a match, label the requirement with the corresponding code. This task required expert knowledge to abstract the main action verbs of the requirement and assign the correct code(s) to it. Because this activity can be challenging due to the polysemy of the main action verb, it was conducted by both annotators. We divided the set of 2755 requirements, used in our qualitative study, into two equal parts. All the requirements of the first part were annotated by the first annotator and reviewed by the second annotator and vice versa. If there was disagreement between annotators, we consulted a financial analyst to reach an agreement using a consensus-based decision-making strategy (Bolander and Sandberg 2013).

We describe below the three activities of the labeling process for requirement DEP0020 in REQS shown in Fig. 2:“IFSIG must store all data for a configurable retention period”. Specifically, (a) we identified that the verb used in the system response is *store*, (b) we detected that *store* matches one of the members of the VerbNet code *keep-15.2*, and (c) we labeled the requirement with the VerbNet code *keep-15.2*.

#### Group requirements (Step 4)

In Step 4 (Fig. 1), we grouped and copied the labeled requirements to different spreadsheets based on their labels. The purpose of having the requirements grouped by label is to make it easier for us to identify common information content among them.

#### Create grammar (Step 5)

**Fig. 3:**
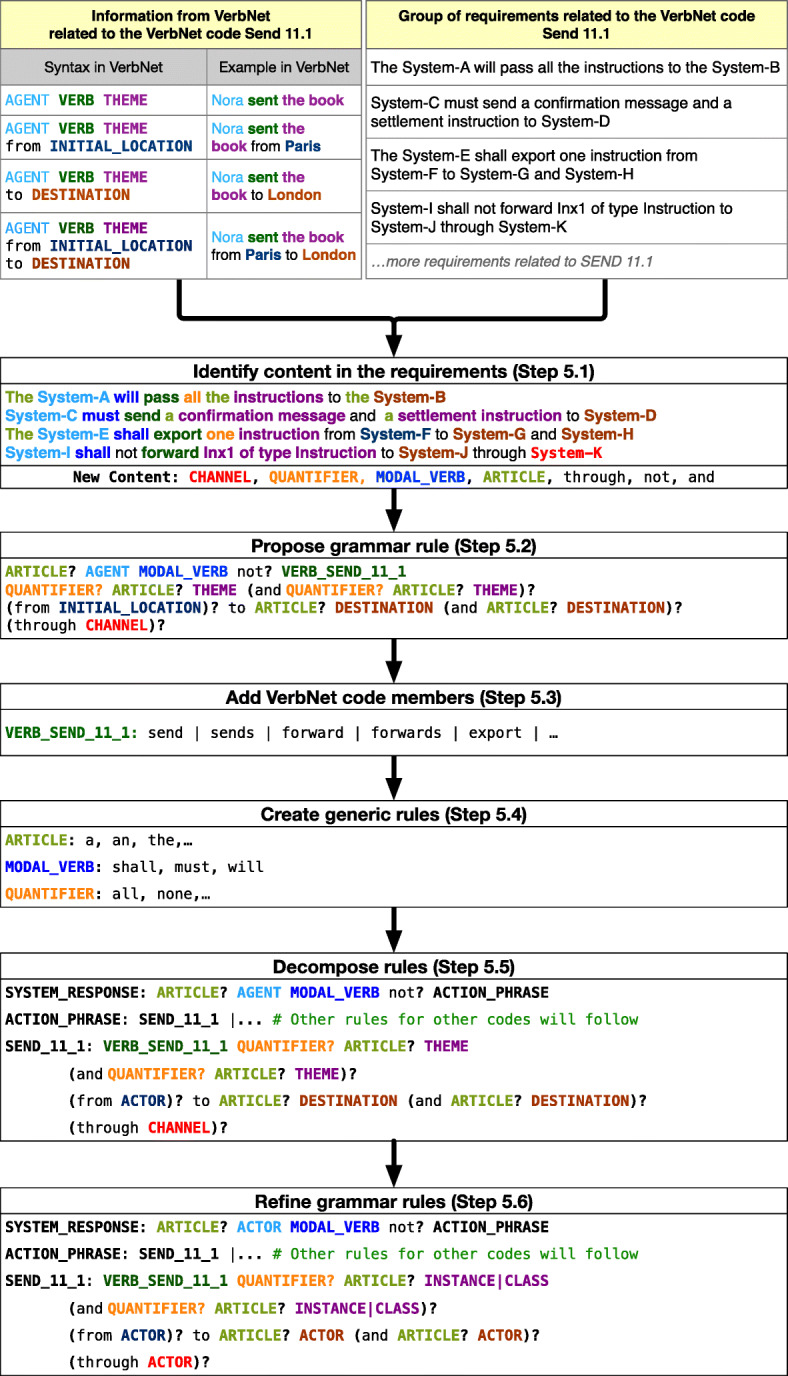
Obtaining CNL grammar rules from requirements related to the VerbNet code *Send 11.1*

In Step 5 (Fig. 1) we created the grammar of Rimay to capture relevant information content from the requirements. Figure 3 shows the steps that we carried out to create grammar rules for the VerbNet code *Send 11.1* (Table 4). The box in the upper-right corner of Fig. 3 shows four examples of requirements related to the VerbNet code *Send 11.1* that will be used to illustrate this step. The same sub-steps (i.e., from 5.1 to 5.6) were carried out for the rest of the codes presented in Tables 4 and 5.

##### Identify content in the requirements (Step 5.1)

In this step we identify semantic roles and keywords in the requirements. VerbNet provides the syntax and the examples that show most of the semantic roles and the keywords (e.g., the prepositions) related to the VerbNet codes in Table 4. For example, the box in the upper-left corner of Fig. 3 shows the syntax and examples related to the VerbNet code *Send 11.1*. The syntax contains the prepositions from and to, and the semantic roles AGENT (a participant that initiates an action), THEME (an entity which is moved by an action, or whose location is described), INITIAL_LOCATION (a place where an event begins or a state becomes true) and DESTINATION (a place that is the end point of an action and exists independently of the event).

In Fig. 3, we use different colors to show the correspondence between the semantic roles and the parts of the requirements that represent the semantic roles. When some content in the requirements was not related to any VerbNet semantic role, we proposed a new semantic role to identify that content. For example, in Step 5.1 of Fig. 3, we proposed the new semantic role CHANNEL to identify the content in the phrase “through System-K”.

##### Propose grammar rule (Step 5.2)

Based on the syntax provided by VerbNet, we defined the order of appearance of the content, and its repetition in Rimay. The symbols ?, ⋆ and + indicate that the users of Rimay can repeat what is before the symbol at most once, any number of times, and at least once, respectively. Step 5.2 in Fig. 3 shows that the grammar rule for the VerbNet code *Send 11.1* contains keywords such as (i) connectors (*and* and *or*), (ii) prepositions shown in the VerbNet syntax (*from* and *to*), (iii) prepositions related to new content (*through*) and (iv) the negation of a modal verb (*not*).

##### Add VerbNet code members (Step 5.3)

We added a complete list of all the members of each VerbNet code related to its corresponding rule. For example, *forward* and *send* are two of the members of the VerbNet code *Send 11.1* that we added to its corresponding rule VERB_SEND_11_1. We also added the conjugated forms of the verbs to the rule (e.g., *forwards*, *sends*).

##### Create generic rules (Step 5.4)

We created the rules related to the generic English grammar, e.g., we created the rules ARTICLE, MODAL_VERB, and QUANTIFIER.

##### Decompose rules (Step 5.5)

We decomposed the grammar rules created in Step 5.2 to make them easier to understand and reuse. For example, we decomposed the example rule in Step 5.2 into three rules: SYSTEM_RESPONSE, ACTION_PHRASE, and SEND_11_1.

##### Refine grammar rules (Step 5.6)

With the help of four financial analysts (including one senior and one mid-career financial analyst), we replaced some of the semantic role names with other ones that were more familiar to both financial analysts and engineers. In our case, financial analysts and engineers working for Clearstream were familiar with the UML (OMG 2017). For example, in the grammar rules SYSTEM_RESPONSE and SEND_11_1 (Step 5.4 in Fig. 3), we chose to replace the role AGENT with ACTOR, because an agent can be represented as an UML actor, i.e., a role played by a human user or a system who initiates and carries out an event or action.

##### Method

The method that we used to create Rimay was iterative and incremental. This means that we first followed Steps 5.1 to 5.6 in Fig. 3 to create the grammar rules related to one of the groups of requirements produced in Step 4 of Fig. 1. Second, we generated a requirements editor using Xtext. Third, we used the generated editor to rephrase the requirements in the first requirements group to test the grammar and its corresponding editor. We tested that our grammar and the editor were expressive enough to allow us to write all the information content for the first group of requirements. If the grammar was not expressive enough, we analyzed and extended the grammar, regenerated the editor and verified the requirements until there were no errors in all the rephrased requirements. For each remaining requirements groups produced in Step 4 (Fig. 1), we repeated Steps 5.1 to 5.6 as performed for the first requirements group. 
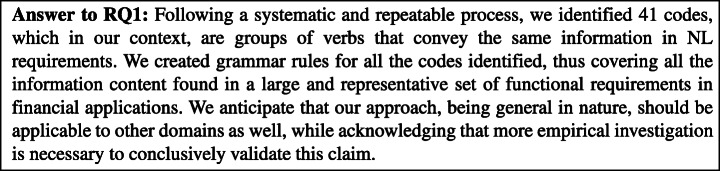


## Controlled natural language for functional requirements

In this section, we describe how a requirement is structured in Rimay in order to answer RQ2: “Given the stakeholders, how can we represent the information content of requirements for financial applications?”.

In recent years, different patterns have been increasingly used by the industry to improve the quality of the requirements. Patterns like EARS (Mavin et al. 2009) and Rupp (Pohl and Rupp 2011) provide general constructs and concepts to specify requirements (Section 2). However, these templates are not amenable to the type of analyses enabling task automation because they allow the introduction of unstructured text. On the other hand, CNLs provide structures with more specialized concepts and constructs, enabling automated analysis. As we report in our recent work, Rimay enables the generation of abstract test cases (Veizaga et al. 2020). Since we could not find any comparable work in the financial domain, we applied Grounded Theory analysis for building Rimay. However, as we explain below, some constructs and concepts of Rimay are inspired by the EARS template.

The rule REQUIREMENT shown in Listing 1 provides the overall syntax for a requirement in Rimay. The rule shows that the presence of the SCOPE and CONDITION_STRUCTURES is optional, but the presence of an ACTOR, MODAL_VERB and a SYSTEM_RESPONSE is mandatory in all requirements.

**Listing 1:**
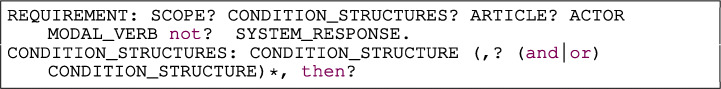
Overall syntax of Rimay

In a requirement, an actor is expected to achieve a system response if some conditions are true. An actor is a role played by an entity that interacts with the system by exchanging signals, data or information (OMG 2017). Moreover, requirements written in Rimay may have a scope to delimit the effects of the system response. One example of a requirement in Rimay is: “ ”. The requirement has a scope (), does not have any conditions, and has an actor () and a system response ().

Throughout this section, we simplify the description of Rimay by considering that the keywords are not case-sensitive. Also, we use grammar rules that are common in English such as MODAL_VERB (e.g., shall, must) and MODIFIER that includes articles (e.g., a, an, the) and quantifiers (e.g., all, none, only one, any). Sections 4.1 and 4.3 will explain the CONDITION_STRUCTURES and SYSTEM_RESPONSE, respectively.

### Condition structures

The grammar rule named CONDITION_STRUCTURE shown in Listing 2 defines different ways to use system states, triggering events, and features, to express conditions that must hold for the system responses to be triggered.

**Listing 2:**
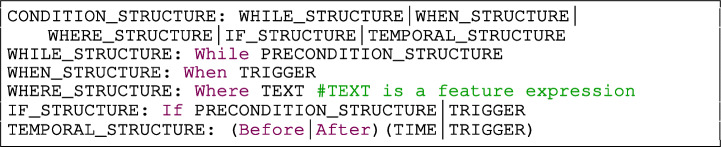
Condition structures

The condition structures WHILE, WHEN, WHERE and IF that we use in our grammar are inspired by the EARS template (Mavin et al. 2009). EARS is considered by practitioners as beneficial due to the low training overhead and the quality and readability of the resultant requirements (Mavin et al. 2016). Additionally, we proposed the rule TEMPORAL_STRUCTURE to be used when the system responses are triggered before or after an event. Below, we describe the types of CONDITION_STRUCTURE used in Rimay:

- The WHILE_STRUCTURE is used for system responses that are triggered while the system is in one or more specific states.
- The WHEN_STRUCTURE is used when a specific triggering event is detected at the system boundary.
- The WHERE_STRUCTURE is used for system responses that are triggered only when a system includes particular features. The features are described in free form using the rule TEXT.
- The IF_STRUCTURE is used when a specific triggering event happens or a system state should be hold at the system boundary before triggering any system responses.

The rule CONDITION_STRUCTURE shown in Listing 2 allows combining condition structures using logical operators. We can, for example, combine the IF and WHEN structures using the operator in the structure “ ” to separate the conditions in which the requirement can be invoked (i.e., the preconditions) and the event that initiates the requirement (i.e., the trigger).

**Listing 3:**
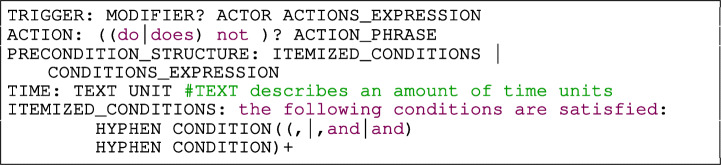
Trigger and precondition structure

**Fig. 4:**
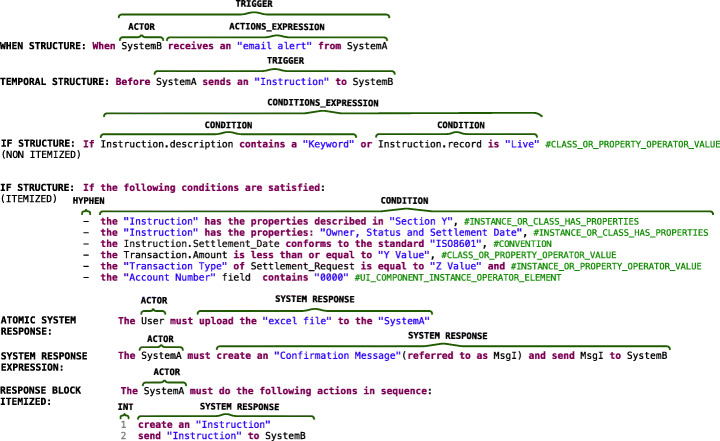
Examples of condition structures and system responses

Figure 4 depicts examples of the WHEN_STRUCTURE, TEMPORAL_STRUCTURE, and IF__STRUCTURE.

Listing 3 shows the grammar rules TRIGGER and PRECONDITION_STRUCTURE referenced by the condition structures in Fig. 4.

The rule TRIGGER in Listing 3 defines that a triggering event is always caused by an ACTOR that performs some actions. The actions performed by the actor are defined by the rule ACTIONS_EXPRESSION which enables the combination of any number of actions using logic connectors to express complex system events. The WHEN_STRUCTURE in Fig. 4 shows an example of a trigger composed of an actor and an action expression: “ ”.

The rule PRECONDITION_STRUCTURE in Listing 3 gives freedom for the users to decide how to describe conditions. The rule ITEMIZED_CONDITIONS (Listing 3) is appropriate for writing long lists of conditions that must evaluate to True. Conversely, the rule CONDITIONS_EXPRESSION (Listing 3) is suitable for only one condition, multiple conditions combined with logical operators, or parentheses that denote priority in the evaluation order of operations. The IF_STRUCTURE in Fig. 4 shows examples of non-itemized and itemized conditions.

### Conditions

In the previous subsection, we introduced the rule PRECONDITION_STRUCTURE to specify conditions. This rule is composed of operands and operators which are described as follows.

#### Operands

The operands are represented by the rules ACTOR, CLASS, PROPERTY, INSTANCE, ELEMENT and TEXT. The meaning of the operands is the same as in the UML (OMG 2017), therefore an *Actor* specifies a role played by the user or another system that interacts with our system. The *Class* represents a domain concept (e.g., Instruction). A *Property* represents the attributes of the *Class*. An *Instance* represents a specific realization of a *Class* and an *Element* is a constituent of a model.

The users of Rimay can use the dot notation to refer to a property of a class, e.g.,“ ”. In the cases where there is only one instance of a class in a requirement, the users do not need to declare any instance. For example, given that in Fig. 4 there is only one instance of an instruction, we used “ ” instead of “ ”.

#### Operators

Rimay uses the following families of operators and its negative forms:

- COMPARE, such as “ ”, “ ”, etc.,
- CONTAINS such as “ ”, “ ”, etc.,
- OTHER OPERATORS such as “ ”

An example of a condition that conforms to Rimay is: “ ”. This condition uses operators of type CONTAINS and COMPARE.

#### Condition rule

The operators and operands defined in the previous subsections are used in the five grammar rules shown in Listing 4 conditions such as the ones shown in Fig. 4.

**Listing 4:**
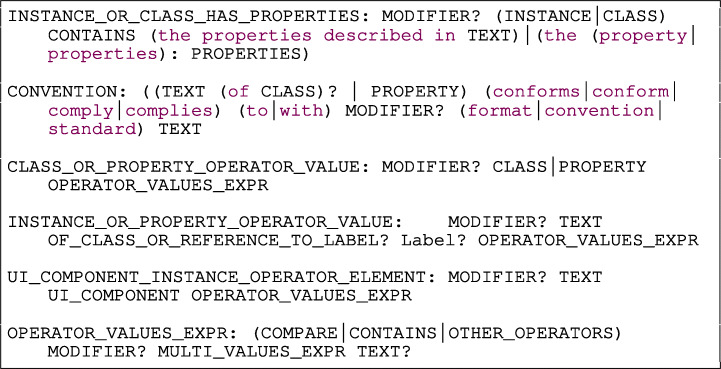
Conditions rules

The types of conditions are described as follows:

1. INSTANCE OR CLASS HAS PROPERTIES evaluates if the instance of a class, or a class itself defines one or more specific properties. The properties can be defined in a document (e.g., “ ”), or directly in the requirement (e.g., “ ”).
2. CONVENTION checks if a property conforms to a format or standard, e.g., “ ”.
3. CLASS OR PROPERTY OPERATOR ELEMENT is a condition composed of an operand-1, an operator and an operand-2. The operand-1 is a reference to a CLASS or PROPERTY. The auxiliary rule OPERATOR VALUES EXPR defines the operator and the operand-2 of the condition, e.g., “ ”. The operand-2 is any type of operand described in Section 4.2.1.
4. INSTANCE OR PROPERTY OPERATOR VALUE is an operand-operator-value condition. The operand is a reference to an INSTANCE or PROPERTY and the value represent any literal or number. An example of this type of condition is: “ ”.
5. UI COMPONENT INSTANCE OPERATOR ELEMENT is a condition composed by an operand-1, operator, and operand-2 for a requirement related to the user interface (UI). The operand-1 is an instance of a UI component identified by a free form TEXT followed by a reference to the type of UI COMPONENT. Rimay contains a list of common UI component types to help the user to create the requirements (e.g., tab, page, bar, field, calendar, checkbox, menu, message). The auxiliary rule OPERATOR VALUES EXPR defines the operator and the operand-2 of the condition. An example that displays this type of condition is: “ ”.

### System response

The rule SYSTEM_RESPONSE in Listing 5 allows the user to express the behavior of the system in two manners using the rules: (a) RESPONSE_BLOCK_ITEMIZED, that is suitable for writing lists of actions; and (b) SYSTEM_RESPONSE_EXPRESSION, that is appropriate for writing one or multiple actions combined with logical operators, or parentheses that denote the priority of the actions. The previous rules include the rule ATOMIC_SYSTEM_RESPONSE and logical operators. Each ATOMIC_SYSTEM_RESPONSE contains an ACTION_PHRASE and optionally, a frequency (e.g., ). Fig. 4 depicts examples of the ATOMIC_SYSTEM_RESPONSE as well as more complex examples, such as SYSTEM_RESPONSE_EXPRESSION and RESPONSE_BLOCK_ITEMIZED.

**Listing 5:**
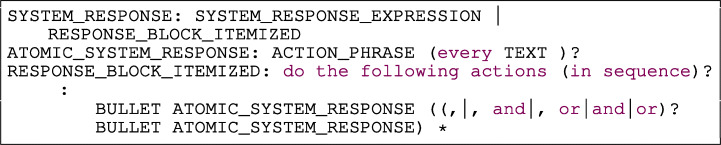
System response

All the types of ACTION_PHRASE rules are available in Appendix A. The rule OBTAIN_13_5_2 in Table 6 is one type of ACTION_PHRASE rule. The column “Grammar Rule Name” shows the name of the grammar rule related to the code *obtain 13.5.2* that we discovered during the qualitative study (Tables 4 and 5). The column “Grammar Rule Summary” describes the syntax of OBTAIN_13_5_2, and the column “Examples” shows requirements that conform to that syntax.

**Table 6:**
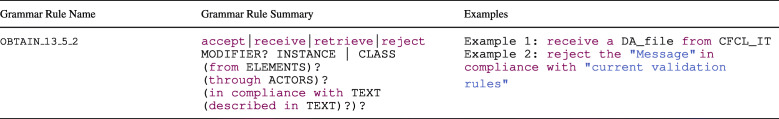
Grammar rule: OBTAIN_13_5_2

#### Rimay editor

**Fig. 5:**
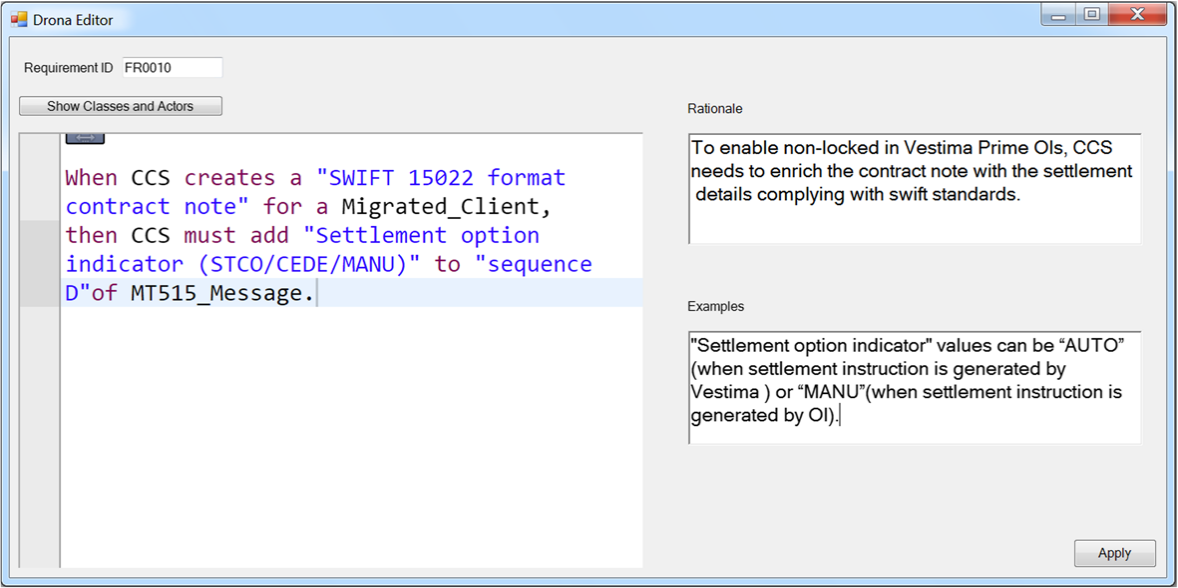
Screenshot of the requirements entry dialog box in the Rimay editor

We developed the Rimay editor using the Xtext language engineering framework (Bettini 2013) which enables the development of textual domain-specific languages. We integrated the Rimay editor into an existing and widely known modeling and code-generation tool: Sparx Systems Enterprise Architect^6^. Enterprise Architect was already being used at Clearstream. In particular, we created a form composed of the Rimay editor, and fields related to key properties of a requirement, such as “Requirement ID”, “Rationale”, and “Examples”. Figure 5

To operationalize our technology-independent grammar (created in Step 5), we need to enhance it with some additional information. In particular, Xtext requires one to declare the name of the language, and further, import reusable terminals such as *INT*, *STRING* and *ID* for the syntax of integers, text, and identifiers, respectively.

The input that we provided to Xtext is an EBNF-like grammar composed of rules that are similar to the ones that we discussed in this section. Xtext automatically generates a web-based editor with the following helpful features (Bettini 2013): (a) syntax highlighting, it allows to have the requirements colored and formatted with different visual styles according to the elements of the language; (b) error markers, when the tool automatically highlights the parts of the requirements indicating errors; and (c) content assist, a feature that automatically, or on demand, provides suggestions to the financial analysts on how to complete the statement/expression. In practice, these features are important to facilitate the adoption of Rimay by financial analysts. The implementation of our grammar and its editor are available online^7^. 
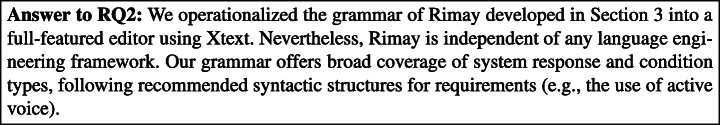


## Empirical evaluation

In this section, we describe a case study that evaluates Rimay developed in Sections 3 and 4. Throughout the section, we follow best practices for reporting on Case Study Research in Software Engineering (Runeson et al. 2012).

### Case study design

As stated in the introduction, our evaluation aims to answer the following research questions:

- **RQ3: How well can Rimay express the requirements of previously unseen documents?**
- **RQ4: How quickly does Rimay converge towards a stable state?**

**Fig. 6:**
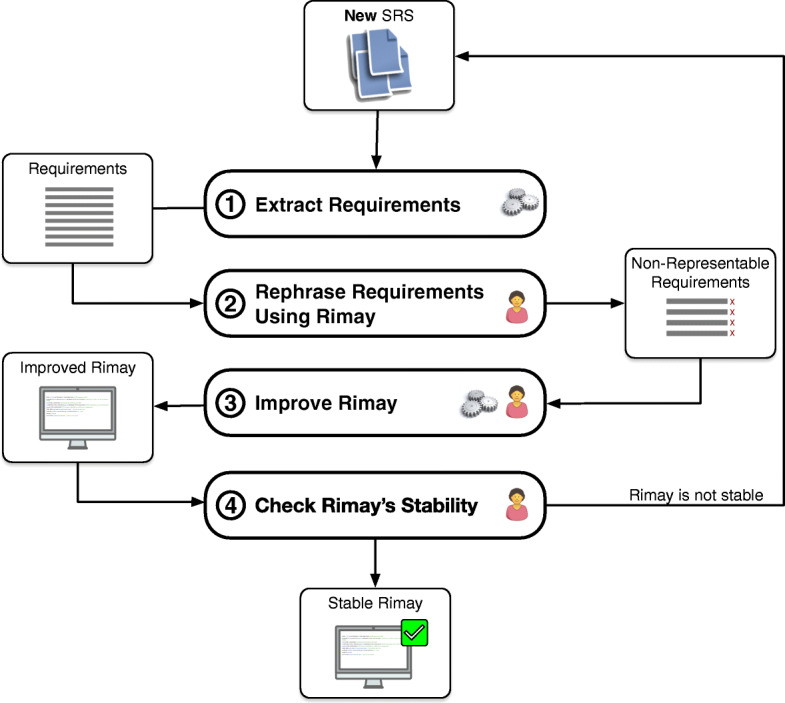
Case study design

Figure 6 shows the iterative process that we follow in order to answer these two questions. To evaluate our approach, we needed to collect new SRSs that had not been used for the construction of Rimay. We applied the four steps presented in Fig. 6 to collect new SRSs and examine the expressiveness and stability of Rimay using them: **(Step 1)** The financial analysts, on an opportunistic basis, gave us a new SRS that we had not seen before; we extracted from the given SRS its NL requirements (“Extract Requirements”, Section 5.1.1). **(Step 2)** We attempted to rephrase the extracted requirements using the rules of Rimay, keeping the intent of the original requirements and ensuring that we did not lose any information content. In this step, we had to keep track of the requirements, if any, that were *non-representable* as well as the causes for such limitations (“Rephrase Requirements Using Rimay”, Section 5.1.2). **(Step 3)** We analyzed the requirements that were marked as *non-representable* and enhanced Rimay to make these requirements *representable* (“Improve Rimay”, Section 5.1.3). **(Step 4)** We checked whether there was a significant change in Rimay’s ability to capture previously unseen content. As we argue in Section 5.4.2, it turned out that with four SRSs (i.e., four iterations of the process in Fig. 6), we were able to reach saturation. At that point, we stopped analyzing more SRSs (“Check Rimay’s Stability”, Section 5.1.4). In the remainder of this section, we will not repeatedly be stating that these four SRSs were collected and analyzed iteratively and in a sequence. Instead, for succinctness, we refer to these four SRSs collectively when it is more convenient to do so.

With regard to our research questions, Step 1 and Step 2 of the process in Fig. 6 answer RQ3, as these two steps provide information about the expressiveness of Rimay, i.e., the requirements that were *representable* or *non-representable* with Rimay. Step 3 and Step 4 of the process address RQ4, as these steps provide information about the improvements necessary for maturing Rimay to a stable state.

#### Extract Requirements (Step 1 of Fig. 6)

In Step 1 of Fig. 6, we extract the requirements from our four new, previously unseen SRSs. These SRSs were selected by senior financial analysts from Clearstream according to the criteria described in Section 3.2. The selected SRSs did not contain any requirement that was already analyzed while building Rimay’s grammar in the qualitative study of Section 3.

#### Rephrase requirements using Rimay (Step 2 of Fig. 6)

This rephrasing activity was performed in an iterative manner. Rephrasing the requirements of the four SRSs into Rimay took four iterations over two months, with each iteration requiring approximately two weeks. Each iteration was interleaved with a face-to-face session of two to three hours with at least six financial analysts (including one senior and one mid-career financial analyst). During the face-to-face validation sessions, the financial analysts checked that the intent of the requirements expressed in Rimay did not deviate from their original intent. A team composed of two annotators (the first and second authors of this article) rephrased the requirements using Rimay. Both annotators rephrased together the first 20% of the requirements (i.e., 92 requirements) in order to internalize a clear procedure for (1) rephrasing a requirement into Rimay and (2) collecting the appropriate data from each requirement (i.e., representability of a requirement and possible causes of non-representability). Having a systematic procedure for rephrasing the requirements alongside the experience that the annotators had already gained while conducting our qualitative study helped ensure the quality of the rephrasing activity over the remaining 80%, i.e., the 368 (460-92) of the requirements that were rephrased by the first annotator.

A requirement can be composed of a scope, pre-conditions, an actor, and a system response. The scope and pre-conditions are optional, but the presence of at least one system response and one actor is mandatory.

Step 2 considers a requirement to be *non-representable* when some information content of the requirement cannot be captured using Rimay. A requirement is considered *representable*, otherwise. A requirement that is *non-representable* is annotated with one of following three causes:

- *Cause 1*. The requirement contains a verb that is not supported by Rimay rules. Therefore, we can either extend a Rimay rule with the verb or create a new rule.
- *Cause 2*. Part of the requirement (excluding the verb) includes information content that is not supported by Rimay. For example, the rule Send 11.1 initially defines the following information content: an AGENT who can move a THEME (e.g., data) from an INITIAL LOCATION to a DESTINATION. If a given requirement involves some information content not considered by Send 11.1 (e.g., the CHANNEL through which the THEME is sent), then we consider that requirement to not be representable according to Cause 2.
- *Cause 3*. The meaning of the requirement is unclear and no financial analyst could clarify it.

#### Improve Rimay (step 3 of Fig. 6)

To improve Rimay, we analyzed the causes for requirements marked as *non-representable*. Concretely, we enhanced Rimay grammar by: (a) creating a new grammar rule when such requirement was marked with *Cause* 1. To create a new grammar rule, we first identified, for each requirement, the codes according to the steps described in Section 3.3.2. The resulting codes were either identified from VerbNet or proposed by us. We then created the grammar rules following the steps described in Section 3.3.5; and (b) updating an existing grammar rule created in Section 3 to include either a new verb of a requirement labeled with *Cause* 1 or missing content of a requirement labeled with *Cause* 2.

Requirements labeled with *Cause* 3 were not addressed in Rimay. We discuss such requirements in Section 6, dedicated to threats to validity.

#### Check Rimay’s stability (step 4 of Fig. 6)

This step verifies whether there was a significant change in Rimay’s capacity to capture the content of previously unseen NL requirements. If there is no significant change, we say that Rimay is stable, and we stop the evaluation process. Otherwise, we iterate over Step 1 to Step 4 using a new SRS until Rimay becomes stable. We refer to the notion of *saturation* to determine the point where Rimay is stable. Saturation is defined mathematically for capturing, in a simple way, when to stop our evaluation. In other words, we stop our evaluation when Rimay is expressive enough to capture all the verbs in the NL requirements of a SRS (i.e., the number of errors due to Cause 1 is zero). In our case study, we reached the saturation point during the evaluation of SRS 4.

### Data collection

**Fig. 7:**
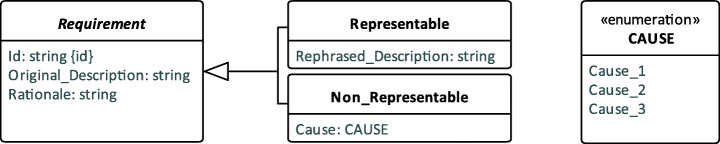
Data model of the collected requirements

We answered RQ3 and RQ4 by collecting data from the execution of the four steps described in Section 5.1. Figure 7 shows the data model of the requirements collected during the empirical evaluation. In our data model, a Requirement has an Id which is a unique code assigned to each requirement, an Original_Description and a Rationale. A requirement is either Representable or Non_Representable. If the requirement is Representable, we recorded its Rephrased_Description. If the requirement is Non_Representable, we recorded the CAUSE (i.e., Cause_1, Cause_2 or Cause_3).

In total, we collected 460 requirements from the four SRSs used in our evaluation. We improved the grammar rules after rephrasing one SRS and assessed the improved grammar on the next.

### Collecting evidence and results

This section describes the execution and the raw data collected from our case study. The case study required the work of two annotators for two months, adding up to approximately 200 person-hours. In Section 5.1.2, we describe how the two annotators performed this task.

Table 7 provides the data for each of the four SRSs. For each SRS, we present the number of requirements that can and cannot be represented using Rimay. For example, the second row of Table 7 shows that 65 (74,7%) out of 87 of the requirements of the first SRS are *representable* in Rimay.

**Table 7:** Percentage of *representable* requirements and frequencies of causes for *non-representable* requirements

Requirements	SRS				Total
	1	2	3	4	
Representable and Non-representable	87	113	192	68	460
Representable	65	96	180	64	405
Non-representable	22	17	12	4	55
Non-representable - Cause 1	11	6	2	–	19
Non-representable - Cause 2	9	8	7	4	28
Non-representable - Cause 3	2	3	3	–	8

Table 7 shows, for the four SRSs, the frequency of the three causes (described in Section 5.1.2) in the requirements labeled as *non-representable*. For example, the second column of Table 7, for SRS 1, shows that for 11 requirements, the verb was not supported by Rimay (*Cause 1*). For nine requirements, some other content was not supported by Rimay (*Cause 2*). Two requirements were unclear and no financial analyst could clarify them (*Cause 3*). In total, 22 out of 87 requirements (25,3%) in SRS 1 were *non-representable.*

Next, we provide examples of *non-representable* requirements for each of the causes described above.

- Cause 1 - SRS 2: “On receipt of a request from System-A to update positions, System-B must recalculate all positions impacted by the confirmed order”. Rimay does not have any grammar rule that has the verb recalculate.
- Cause 2 - SRS 1: “When the Market Calendar does not exist in the System, the System must add a record about the missing Market Calendar to the exception log”. The grammar rule Mix-22.1-2, that contains the verb “add” does not support the following information content “about missing market calendar”.
- Cause 3 - SRS 3: “System-A must be able to process System-B${}^{\prime }$s instructions with input media INPUT”. The requirement is vague since the verb “process” is not precise enough (Femmer et al. 2014).

Finally, we improved Rimay by addressing the *non-representable* requirements labeled with Causes 1 and 2, as explained in Section 5.1.3.

#### Coding results

Tables 8 and 9 show the codes and their verb members identified during our empirical evaluation. Recall from Section 3 that a code represents a group of verbs that convey the same information in NL requirements. The structures of Tables 8 and 9 are the same as the structures of Tables 4 and 5 reporting the coding results of our qualitative study discussed in Section 3.

**Table 8:** VerbNet codes identified during our empirical evaluation

Codes		Members
Class name	Hierarchy Level	
begin	55.1-1	start
∗establish	55.5-1	establish
other_cos	45.4	reverse
remove	10.1	deduct
∗search	35.2	search
send	11.1	export
∗stop	55.4	stop
use	105	use
Total: 8

**Table 9:** Codes proposed during our empirical evaluation

Codes	Members
∗calculate	calculate, recalculate
∗split	split
∗subscribe	subscribe
∗upload	upload
update	set
Total: 5	

Seven out of 13 codes in Tables 4 and 5 were found during our empirical evaluation. We placed the symbol “*” before the seven new codes to differentiate them from the codes that we had already identified in the qualitative study. For each new code, we created a new grammar rule. Considering that, in total, we found 48 codes during the qualitative study and the empirical evaluation, the seven (14,6%) new codes found in the empirical evaluation did not prompt drastic modifications to Rimay.

### Analysis of collected data

In this section, we analyze the collected data and answer RQ3 and RQ4.

#### Performance of Rimay on previously unseen SRSs (RQ3)

Table 7 shows that 405 out of 460 requirements (88%) across all four SRSs can be expressed using Rimay. For SRS 1, we use the version of Rimay resulting from our qualitative study while, for the following SRSs (second to fourth), we use a version of Rimay that includes the improvements made based on the previous SRS(s).

With regard to SRS 1, we note that we found five occurrences of a new verb, “use”, which we had not encountered during our qualitative study. The relatively low expressiveness in this first SRS is largely explained by the high frequency of appearance of this single verb. As one can see from Table 7, most requirements can be represented in Rimay across all SRSs. The improvements to the expressiveness of Rimay are brought about by small changes to Rimay. In other words, while the expressiveness of our grammar did improve as the result of analyzing more SRSs, we did not have to make major changes to the grammar. Our changes involved only the introduction of a few new verbs (as shown in Tables 8 and 9), and the enhancements of a small number of grammar rules created during our qualitative study (Section 3).

The most common causes for a requirement to be *non-representable*, in order of prevalence, are *Cause 2* with 28 occurrences (50.9%), followed by *Cause 1* with 19 occurrences (34.5%), and, finally *Cause 3* with 8 occurrences (14.5%). We conjecture that the main reason why *Cause 2* turns out to be the most frequent cause is that VerbNet – the lexicon we use for deriving our grammar rules – is domain-independent and may not contain certain information content that is specific to the financial domain. During our qualitative study, we identified some new content and extended the grammar rules accordingly. For example, the syntax for the rule Send_11.1 in VerbNet specifies that an AGENT can move a THEME (e.g., data) from an INITIAL_LOCATION to a DESTINATION. Then, during the qualitative study, we identified new information content such as the temporal structure (e.g., “Before 1h00 CET”) used at the beginning of requirements. Furthermore, in the evaluation, we identified extra information content such as a valid channel to send the THEME (e.g., a subsystem that encrypts the data). 
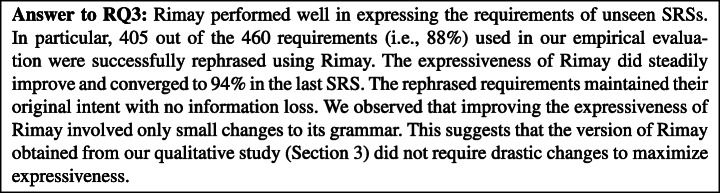


#### Ensuring the stability of Rimay (RQ4)

We refer to the notion of *saturation* to determine the point in our evaluation where we have been through enough SRSs to be confident that the updated version of Rimay is as expressive as possible to specify requirements for the financial domain. To determine if a statistically significant change is observed in the percentage of *representable* requirements, we conduct z-tests for differences in proportions of representability across different SRSs.

##### Saturation

Usually, saturation is reached in a qualitative study when “no new information seems to emerge during coding, i.e., when no new properties, dimensions, conditions, actions/interactions, or consequences are seen in the data” (Glaser 2006). In our evaluation, the saturation point is reached when all the verbs analyzed in a SRS are already considered by Rimay (i.e., when *Cause 1* is not triggered). Specifically, as shown in Table 7, SRS 4 was the only SRS where no requirement was classified as *non-representable* due to *Cause 1*.

As can be seen from Table 7, the increment in the percentage of requirements that can be written in Rimay is tangible evidence that the changes made to Rimay were beneficial (although not extensive).

##### Z-test.

The z-test is a standard statistical test used for checking the difference between two proportions (Dietterich 1998). We run one-tailed z-tests to check if the proportion (*p*_1_) of *representable* requirements in one SRS (SRS *i*) is larger than or equal to the proportion (*p*_2_) of *representable* requirements in another SRS (SRS *j*) analyzed thereafter. Our null and alternative hypotheses are as follows:

$$H_{0} : p_{1} \geq p_{2} $$

$$H_{1} : p_{1} < p_{2} $$

- *H*_0_ : The percentage of *representable* requirements does not increase from SRS *i* to SRS *j*.
- *H*_1_ : The percentage of *representable* requirements increases from SRS *i* to SRS *j*.

Each sample contains more than 30 independent data points and, though sample sizes are not equal, they are not drastically different, thus allowing the use of z-tests (Zikmund et al. 2013). In total, we run six z-tests, at a level of significance of 0.05. The SRS pairs covered by these tests, alongside their corresponding proportions, are shown in Table 10. For example, the first row of Table 10 shows the input for performing a z-test over the (SRS 1, SRS 2) pair. SRS 1 contains 65 requirements that are *representable* with Rimay out of 87 requirements, and SRS 2 contains 96 requirements that are *representable* with Rimay out of 113 requirements.

**Table 10:** Z-tests inputs

Test	Input				
	Document Pair	Sample	Sample	Representable	Representable
	SRS *i*, SRS *j*	Size in	Size in	Requirements	Requirements
		SRS *i*	SRS *j*	in SRS *i*	in SRS *j*
				(*p*_1_)	(*p*_2_)
1	SRS 1, SRS 2	87	113	65	96
2	SRS 1, SRS 3	87	192	65	180
3	SRS 1, SRS 4	87	68	65	64
4	SRS 2, SRS 3	113	192	96	180
5	SRS 2, SRS 4	113	68	96	64
6	SRS 3, SRS 4	192	68	180	64

The z-scores and p-values for the z-tests are shown in Table 11. We conclude that the null hypothesis, *H*_0_, is *rejected in the first five z-tests*. Therefore, there is significant evidence to claim that proportion *p*_1_ is less than proportion *p*_2_ at the 0.05 significance level for the first five document pairs. Concretely, this means that the proportion of *representable* requirements in SRS 2, SRS 3, and SRS 4 are significantly better than that of SRS 1. Similarly, the proportion of *representable* requirements in SRS 3 and SRS 4 are significantly better than that of SRS 2. However, the null hypothesis *cannot be rejected in the last z-test*. Therefore, the proportion of *representable* requirements in SRS 4 is not significantly better than that of SRS 3. We therefore concluded our analysis of new SRSs after completing SRS 4.

**Table 11:** Z-test results

Test	Document Pair	z	*p* − *value*
	SRS *i*, SRS *j*		
1	SRS 1, SRS 2	− 1,81	0,03
2	SRS 1, SRS 3	− 4,50	3,35 E-06
3	SRS 1, SRS 4	− 3,21	6,67 E-4
4	SRS 2, SRS 3	− 2,53	0,01
5	SRS 2, SRS 4	− 1,86	0,03
6	SRS 3, SRS 4	− 0,11	0,46


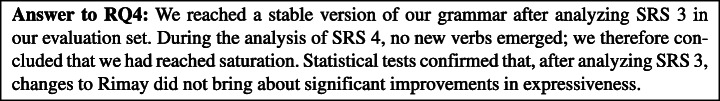


## Threats to validity

In the following subsections, we analyze potential threats to the validity of our empirical work according to the categories suggested by Wohlin et al. (2012) and adapted by Runeson et al. (2012) for case studies in software engineering.

### Construct validity

Construct validity reflects to what extent the operational measures that are studied really represent what the researcher has in mind and what is investigated according to the research questions (Runeson et al. 2012).

We measured the percentages of the requirements that can be represented with Rimay according to the grammar rules we identified. If the criteria that we used to assess whether a requirement is *representable* are incomplete or too strict, this could constitute a threat. We therefore proposed three criteria (named *Causes*) that alleviate the risk of introducing inadequate information content into Rimay. We analyzed the *Causes* of the requirements marked as *non-representable* in order to enhance the Rimay grammar by (a) creating new grammar rules (i.e., *Cause 1*); (b) updating grammar rules to include some missing content (i.e., *Cause 1* and *Cause 2*), and (c) not considering incomplete, ambiguous or unclear information content (i.e., *Cause 3*). *Cause 1* and *Cause 2* are meant to capture missing parts that need to be included in the Rimay grammar. On the other hand, *Cause 3* focuses on the requirements that describe incorrect information content that we do not want to include in Rimay. To be sure that no important information was excluded from Rimay, we looked at the eight *non-representable* requirements labelled with *Cause 3* (Table 7) with the senior financial analysts from Clearstream, who agreed with our decision to discard them.

A second threat to construct validity is related to potential biases in the interpretation of requirements and the application of the qualitative codes while conducting Step 3 (i.e., Label Requirements) in Section 3. Ideally, to prevent biases in the coding process, one could have involved third parties in carrying out the step. However, we did not do so for two main reasons: (1) the confidentiality agreement with our industrial partner did not allow us to share the requirements with external parties, and (2) it was infeasible to identify third parties that had the specialized knowledge required for the coding process driven by linguistic resources, notably, VerbNet and WordNet. Despite not having third parties involved in this activity, we were able to mitigate potential biases and ensure the quality of the results by primarily relying on linguistic resources (VerbNet and WordNet, as noted above). Furthermore, whenever we were unable to conclusively interpret a requirement, we escalated the case to our collaborating financial analysts for deciding about the interpretation.

### Internal validity

Internal validity is of concern when causal relations are examined (Runeson et al. 2012).

The results and the conclusions of our study strongly rely on two key activities that were performed manually: (1) the identification of codes (carried out by using protocol coding) and their members, and (2) the transformation process of requirements into Rimay. This can represent an important threat to the internal validity of our study. To mitigate biases, these two activities were systematically performed by a pair of researchers (the first and second author of this article). Afterward, a third researcher (the third author of this article) reviewed and challenged some of the results of these activities. We finally improved steps (1) and (2) upon reaching an agreement between these three researchers.

Another threat to the internal validity is related to the assumption that all the requirements in SRSs should be used to create Rimay. If all the requirements in SRSs are used, incomplete and unclear requirements might be easily misinterpreted and as a consequence, incorrect information content might be included in Rimay. To tackle this threat, in Step 2 “Rephrase Requirements Using Rimay” (Fig. 6), we first classified as *non-representable due to Cause 3* the requirements that contained either incomplete or unclear information and we then discarded those requirements.

### External validity

External validity is concerned with the extent to which it is possible to generalize the study findings, and to what extent the findings are of interest to other people outside the investigated case (Runeson et al. 2012).

The generalizability of our results is subject to certain limitations. For instance, by design, Rimay is focused on and applicable to functional requirements in the financial domain. In addition, overfitting is a potential threat because of the similarity in background among the eight financial analysts involved in the creation and validation of Rimay (Section 3.2). To mitigate this threat, we designed our analysis procedure (Section 3.3) by minimizing reliance on domain-specific terms from the financial domain. In particular, the fact that our procedure is rooted in domain-independent lexical resources (i.e., VerbNet and WordNet) significantly reduces the risk of overfitting. For this reason, we conjecture that many of our findings can be generalized to information systems in other similar domains.

A company who would want to reuse Rimay should first assess how complete Rimay is in capturing all their requirements; second, it should identify the changes required to our methodology to achieve a satisfactory degree of completeness in their given domain.

### Reliability validity

Reliability validity is concerned with the extent to which the data and the analysis are dependent on the specific researchers involved (Runeson et al. 2012). In order to achieve acceptable reliability, research steps must be repeatable, i.e., other researchers have to be able to replicate our results (Badampudi et al. 2016).

It is impossible to build a CNL that is able to represent all software requirements, and as we already acknowledged, some requirements could not be represented with Rimay. The main issues that may constitute a threat to reliability are related to how we built our CNL to be as expressive as possible. To mitigate this threat, we described in details the steps of our qualitative study and empirical evaluation following a systematic process. This process was performed by the first and second authors and monitored by the other authors of the article.

## Practical considerations

In this section, we present some practical considerations for the different audiences who may be interested in the work reported in this article. These considerations are based on both our experience and our interactions with our industrial partner.

### Considerations for CNL builders

The creation of a language editor entails a significant level of effort because there are many tasks to support, such as auto-completion and syntax highlighting. Mature language engineering frameworks make these tasks less complicated or even fully automated. For instance, we used Xtext to generate a basic editor based only on the grammar of Rimay. For us, the most challenging part of defining a grammar was to understand how to model nested expressions. The effort to customize the generic behavior of the editor generated by Xtext should be considered. In our case, we use the generic editor for our evaluation, but we are in the process of customizing the editor to further improve usability. In particular, we are simplifying the error messages shown by Rimay’s editor, since they are difficult to understand for people without technical knowledge.

### Considerations for companies investing into a CNL

Additional effort is to be anticipated for integrating a CNL with existing software development tools. In our case, our industrial partner uses Sparx Systems Enterprise Architect for modeling UML Use Case, Class, and Activity Diagrams. A key consideration for our partner was therefore to be able to reference (from requirements) the elements of UML models in Enterprise Architect. To provide such functionality, Rimay’s editor dynamically tracks the model elements that need to be referenceable from requirements. This allows Rimay’s editor to provide context-sensitive auto-completion assistance as analysts type in their requirements. Furthermore, if an analyst introduces in a requirement an element that does not already exist in the UML model, our editor will notify the analyst, asking whether the new element should be added to the UML model.

Whether an organization should invest into a CNL for requirements also depends on how requirements are elaborated and used within the organization. Generic text editing tools may suffice for analysts working on small projects. In our case, the types of projects our industrial partner is engaged in justified the construction of a CNL; the projects are not only large and complex but also involve multiple analysts from geographically dispersed locations. Systematic requirements writing practices that help mitigate incompleteness and ambiguity are thus key for our partner. In addition, organizations are interested in extracting accurate information from the requirements as a prerequisite step for automating such tasks as consistency checking between models and (textual) requirements, as well as generating test cases from requirements. Working toward such automation objectives would be very difficult without structured requirements, thus further justifying investment into a CNL. In more recent work (Veizaga et al. 2020), our partners recognized that generating acceptance criteria exclusively from models would miss critical information that is available only in NL requirements. In that work, we elaborate on how acceptance-criteria-relevant information in NL requirements expressed via Rimay can be used for enriching requirements models and subsequently obtaining more precise and complete acceptance criteria.

### Extending Rimay to other domains

In this paper, we focused on the financial domain. However, Rimay may be adapted for use in other domains. We recommend the following steps to adapt Rimay to a given organization:

1. *Select requirements.* The organization selects functional requirements that are representative of commonly used conditions and action phrases.
2. *Rephrase requirements using Rimay.* The organization first rewrites the requirements selected in the previous step using Rimay, and second, labels each *non-representable* requirement with one of the three causes described in Section 5.1.2. Domain experts must ensure that the intents of the requirements written in Rimay do not deviate from the original ones.
3. *Improve Rimay.* For each *non-representable* requirement, the organization should enhance Rimay’s grammar by either updating the existing grammar rules or creating new ones. The organization must follow the methodology described in Section 5.1.3 to perform this step.
4. *Generate and integrate Rimay’s editor.* Once the organization has enhanced Rimay’s grammar to support previously *non-representable* requirements, it generates and integrates the extended version of Rimay’s editor into the modeling and development tool used within the organization, if available. If the editor is created using the Xtext language engineering framework, it can be used as an Eclipse-based plugin or integrated into web applications.

The time required for an organization to extend Rimay is difficult to estimate since doing so depends on several factors: (1) the number of requirements to be rephrased using Rimay, (2) the degree of access to engineers who know Rimay’s methodology and have a background in language engineering, and (3) sufficient access to domain experts.

Since there is currently no extension of Rimay, to gain insights into the time required to extend Rimay, we discuss relevant aspects of the evaluation and refinement of Rimay presented in Section 5. The evaluation of Rimay included (1) a set of 460 functional requirements, (2) two engineers (first two authors of this article), and (3) six domain experts. The entire evaluation and refinement process required 200 hours from the engineers and eight hours from domain experts over a span of two months (Section 5.3). The (approximate) distribution of effort observed across the four steps of our approach was as follows: Select requirements (10%), Rephrase requirements using Rimay (60%), Improve Rimay (25%), and Generate and integrate Rimay’s editor (5%).

## Conclusions

In this article, we proposed a rigorous methodology to define controlled natural languages (CNLs) for requirements specifications. We applied this methodology to develop a CNL, which we named Rimay, for expressing functional requirements in the financial domain. Rimay’s grammar was derived from a qualitative study based on the analysis of 2755 requirements from 11 distinct projects. In this qualitative study, we identified the information content that financial analysts should account for in the requirements of financial applications.

We conducted an empirical evaluation of Rimay in a realistic setting. This evaluation measured the percentage of requirements that can be represented using Rimay. We observed that, on average, 88% of the requirements that we evaluated in our case study (405 out of 460) could be expressed using Rimay. Additionally, we analyzed how quickly Rimay would converge and stabilize to even higher percentages when refined after each new requirements specification was analyzed.

To a large extent, because it was specifically designed to be domain independent, we believe that Rimay can address the broader domain of data-intensive information systems. That said, future investigations remain necessary to determine whether and how Rimay can be specialized for other domains.

While CNLs and requirements patterns have generated a lot of attention in recent years as a vehicle for improving the quality of natural-language requirements, to our knowledge, no previous study has proposed and evaluated a CNL based on a qualitative analysis of a large number of industrial requirements and following a systematic process using lexical resources. A significant portion of this article was dedicated to developing and discussing such a systematic process with the goal of making this process repeatable; this way, other researchers and practitioners interested in developing their own CNLs can benefit from our proposed process and possibly even use Rimay as a starting point.

For future work, we intend to conduct a user study on the usefulness of Rimay. This would assess in a more conclusive manner whether financial analysts benefit from using Rimay for specifying functional requirements.

## Figures and Tables

**Table 12:**
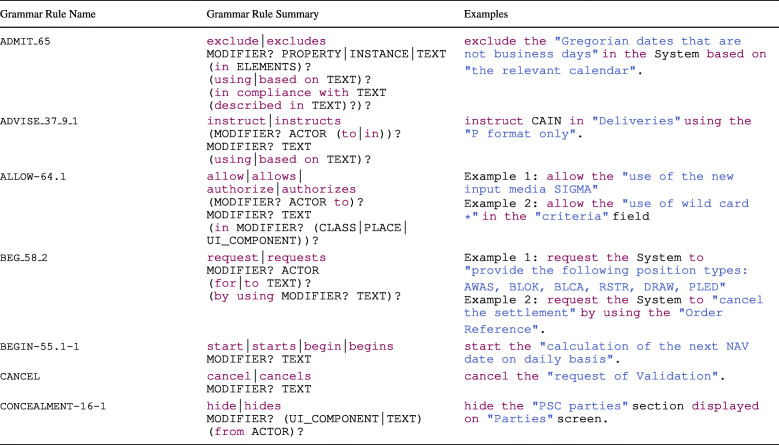

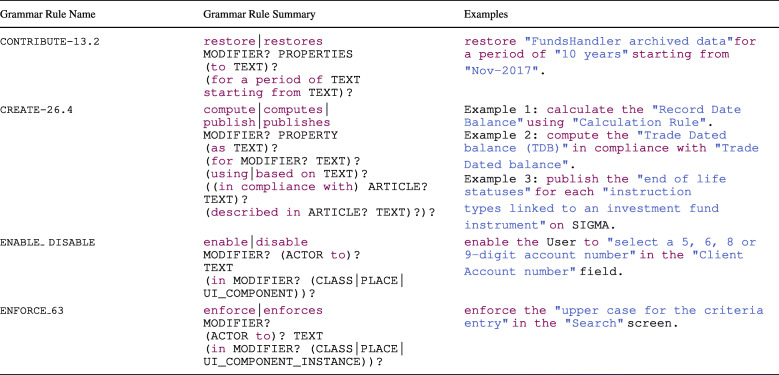

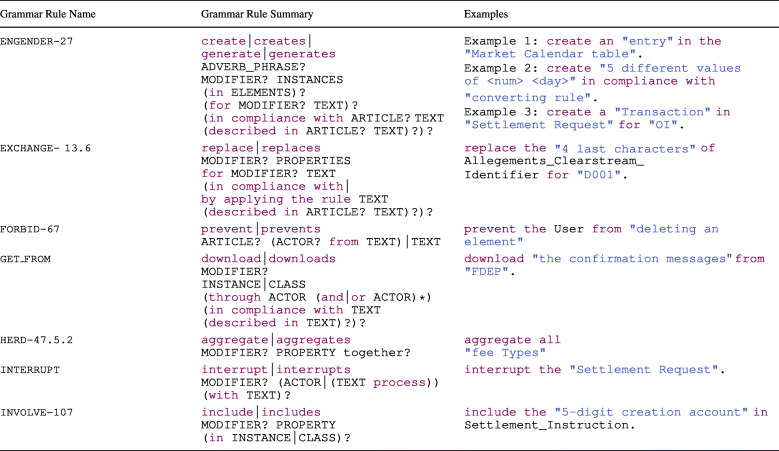

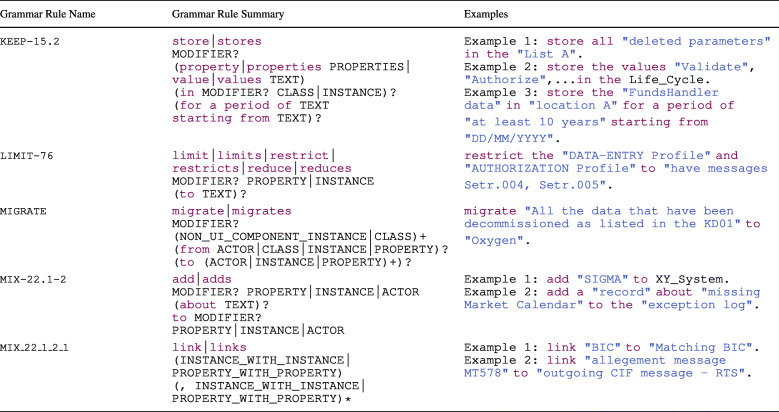

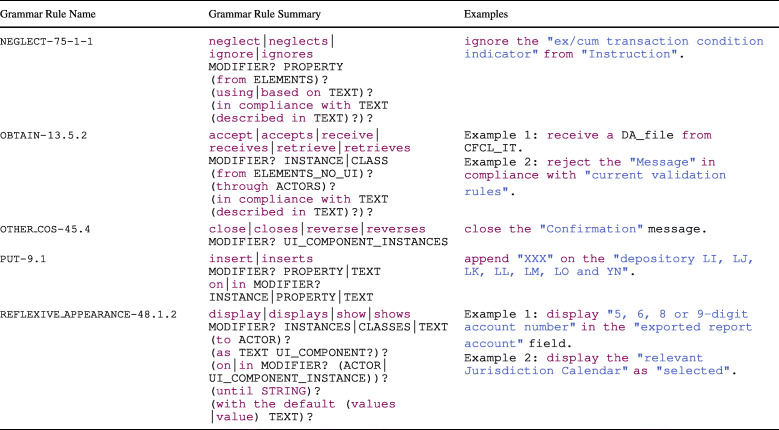

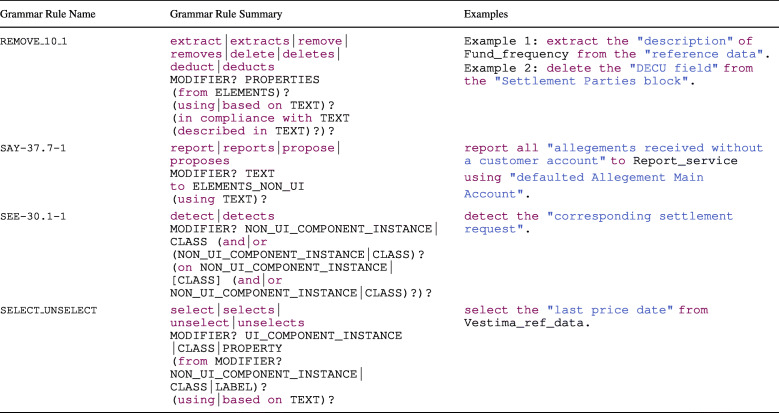

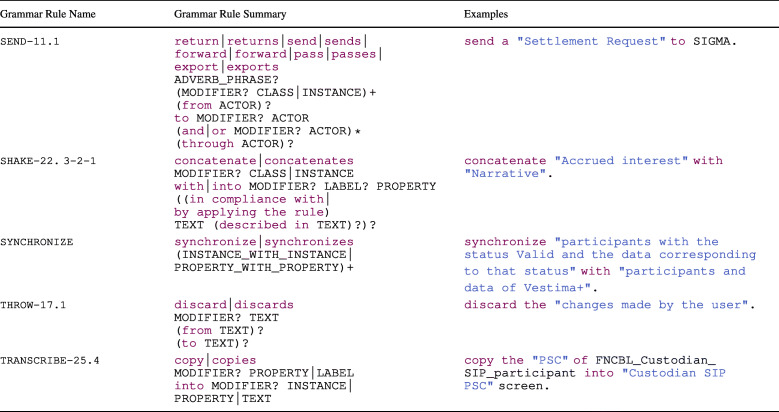

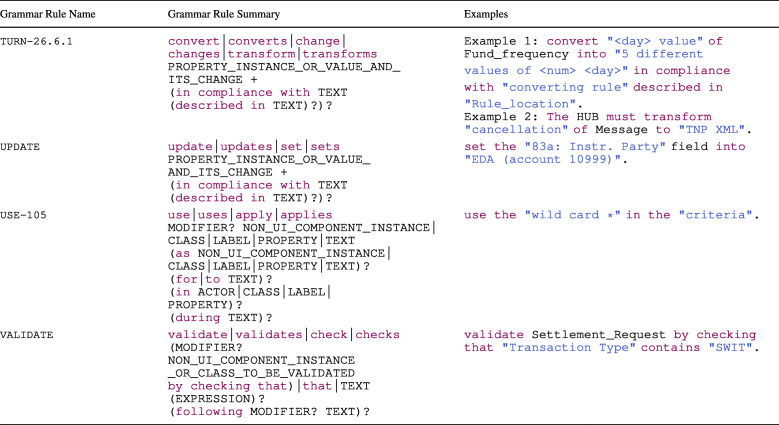
Types of action phrase rules in Rimay (from Qualitative Study)

**Table 13:**
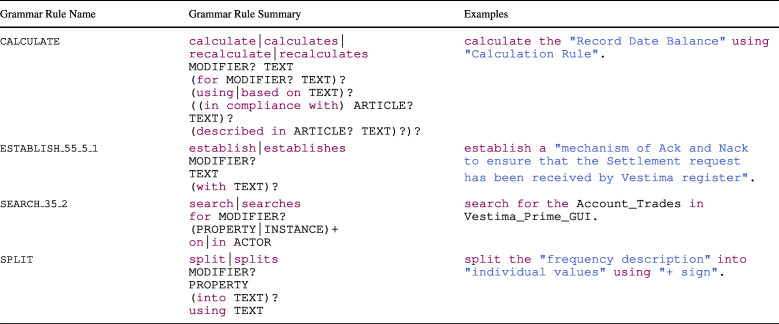

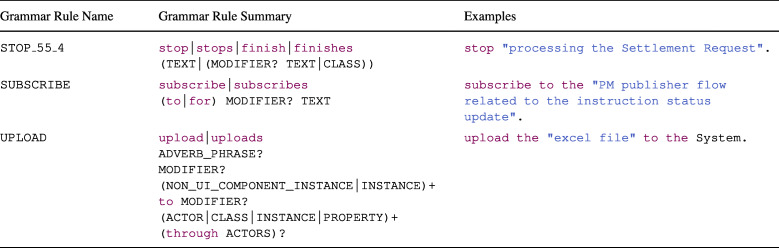
Types of action phrase rules in Rimay (from Empirical Evaluation)

## Appendix A: Action Phrases in Rimay

Tables 12 and 13 show the name, summary, and examples of the Rimay grammar rules related to action phrases. Table 12 displays the rules built during the qualitative study and Table 13 depicts the rules created in the empirical evaluation.

## Footnotes

1 https://verbs.colorado.edu/verbnet/ (last access on 26 January 2021)

2 https://wordnet.princeton.edu/ (last access on 26 January 2021)

3 https://www.eclipse.org/Xtext/ (last access on 26 January 2021)

4 https://poi.apache.org/ (last access on 26 January 2021)

5 https://www.nltk.org/ (last access on 26 January 2021)

6 https://sparxsystems.com/products/ea/ (last access on 26 January 2021)

7 https://gitlab.uni.lu/aveizaga/dsl_rimay/ (last access on 26 January 2021)
